# Disrupting glioblastoma networks with tumor treating fields (TTFields) in in vitro models

**DOI:** 10.1007/s11060-024-04786-0

**Published:** 2024-08-01

**Authors:** Steffen Schlieper-Scherf, Nils Hebach, David Hausmann, Daniel D. Azorín, Dirk C. Hoffmann, Sandra Horschitz, Elena Maier, Phillip Koch, Matthia A. Karreman, Nima Etminan, Miriam Ratliff

**Affiliations:** 1 Department of Neurosurgery, University Hospital Mannheim, University of Heidelberg, Mannheim, Germany; 2 Clinical Cooperation Unit Neurooncology, German Cancer Research Center (DKFZ), German Cancer Consortium (DKTK), Heidelberg, Germany; 3 Neurology Clinic and National Center for Tumor Diseases, University Hospital Heidelberg, Heidelberg, Germany; 4 Central Institute of Mental Health, University of Heidelberg/Medical Faculty Mannheim, Mannheim, Germany; 5 Hector Institute for Translational Brain Research (HITBR gGmbH), Mannheim, Germany

**Keywords:** Glioma, Cancer neuroscience, Tumor microtubes, Cancer cell network, TTFields

## Abstract

**Purpose:**

This study investigates the biological effect of Tumor Treating Fields (TTFields) on key drivers of glioblastoma’s malignancy—tumor microtube (TM) formation—and on the function and overall integrity of the tumor cell network.

**Method:**

Using a two-dimensional monoculture GB cell network model (2DTM) of primary glioblastoma cell (GBC) cultures (S24, BG5 or T269), we evaluated the effects of TTFields on cell density, interconnectivity and structural integrity of the tumor network. We also analyzed calcium (Ca^2+^) transient dynamics and network morphology, validating findings in patient-derived tumoroids and brain tumor organoids.

**Results:**

In the 2DTM assay, TTFields reduced cell density by 85–88% and disrupted network interconnectivity, particularly in cells with multiple TMs. A “crooked TM” phenotype emerged in 5–6% of treated cells, rarely seen in controls. Ca^2+^ transients were significantly compromised, with global Ca^2+^ activity reduced by 51–83%, active and periodic cells by over 50%, and intercellular co-activity by 52% in S24, and almost completely in BG5 GBCs. The effects were more pronounced at 200 kHz compared to a 50 kHz TTFields. Similar reductions in Ca^2+^ activity were observed in patient-derived tumoroids. In brain tumor organoids, TTFields significantly reduced tumor cell proliferation and infiltration.

**Conclusion:**

Our comprehensive study provides new insights into the multiple effects of Inovitro-modeled TTFields on glioma progression, morphology and network dynamics in vitro. Future in vivo studies to verify our in vitro findings may provide the basis for a deeper understanding and optimization of TTFields as a therapeutic modality in the treatment of GB.

**Supplementary Information:**

The online version contains supplementary material available at 10.1007/s11060-024-04786-0.

## Introduction

Gliomas, particularly glioblastomas (GB), are the most common primary malignancies of the central nervous system [1]. Glioma cells have a notorious capability to extensively infiltrate neighboring healthy tissue. They are also remarkably resistant to conventional therapies. Together, these features contribute to the almost inevitable recurrence of these tumors, even after exhaustive approaches such as maximal safe resection, radiotherapy and temozolomide chemotherapy.

A distinctive feature of glioma cells is their possession of neurite-like membrane protrusions called tumor microtubes (TMs). These TMs play a pivotal role in various processes, including brain invasion, proliferation and long-range intercellular connectivity, forming a functional network that enables intricate multicellular communication [2–7]. In particular, this tumor cell network receives neuronal input that actively influences tumor progression [3, 4].

This intricate network provides a protective buffer against the cytotoxic effects of radiation and temozolomide chemotherapy [2, 8]. Cells integrated into the tumor cell network through TM-mediated gap junctions demonstrate increased resilience to cell death during treatment, whereas tumor cells existing in isolation are more susceptible to undergo apoptosis [2, 8]. Our recent studies have unveiled the existence of a subpopulation of highly active tumor cells within this network. Network analysis has revealed specific tumor cells characterized by periodic calcium (Ca^2+^) oscillations [7]. These particularly active tumor cells are primarily situated within hubs of the small-world and scale-free GBC networks and constantly activate the rest of the network cells in a pacemaker-like manner [7]. Building upon this observation, our fundamental hypothesis is that interfering with the preservation of the glioma tumor network might offer a new avenue for glioma therapy.

The use of Tumor Treating Fields (TTFields) therapy has shown promising results in prolonging disease-free intervals and overall survival in a phase III clinical trial conducted in GB patients [9]. For the treatment of GB, TTFields uses a non-invasive medical device to generate low intensity (1–2 V/cm), intermediate frequency (200 kHz) alternating electric fields. The efficacy of this treatment modality depends on the dosage, frequency, and precise location of the tumor [10–13]. However, the exact effect of this treatment on tumor biology remains a subject of controversy. Given the inherent susceptibility of many cellular structures to electric force due to their composition of charged elements [14–16], the intricate mechanisms underlying the effects of TTFields therapy on tumor biology warrant further exploration and clarification.

TMs are primarily composed of filamentous actin (F-actin), mitochondria, microvesicles and gap junctions containing Connexin 43 [2–6]. Gap junctions establish cytoplasmic connections between neighboring cells, facilitating the exchange of small molecules, including Ca^2+^ [2, 5, 17]. This gap junction-mediated intercellular communication can lead to the generation of microcurrents between cells [7], rendering it a potential target for electric fields.

Consequently, we sought to investigate how the use of Inovitro devices designed to model TTFields in vitro affects TM structures and TM-dependent tumor cell network functionality.

## Material and methods

### Glioblastoma stem cell lines, patient-derived GB tumoroids and GBCs growing in tumor organoids

Primary human glioblastoma stem-like cell lines (GBCs: S24, BG5, T269, P3) [18, 19] were cultured under serum-free, non adherent, stem-like conditions in DMEM-F12 medium (#11,330–032, Life Technologies, part of Thermo Fisher Scientific, Waltham, Massachusetts, USA) with B27 supplement (#17504044, Life Technologies), insulin (5 μg/ml; #I9278, Sigma, part of Merck, Darmstadt, Germany), heparin (5 μg/ml; #H4784, Sigma), epidermal growth factor (20 μg/ml EGF; #PHG0311, Life Technologies), and fibroblast growth factor (20 μg/ml basic FGF; #PHG0021, Life Technologies). All GBCs used were diagnosed as glioblastoma, IDH wild-type and their origins confirmed via Methylation EPIC Array (#WG-317–1003, Illumina, San Diego, California, USA). Authenticity and absence of contamination were regularly checked (Multiplexion GmbH, Heidelberg, Germany).

For 2DTM, GBCs were plated after singularization with Accutase (#1110501, Thermo Fisher Scientific) and maintained in high glucose medium (HGM; 50 mM glucose; #G7021-1 KG, Sigma) as previously described [7].

The choice of the terms “tumoroids” and “brain tumor organoids” is intended to clarify the origin and composition of 3D GB models. The term “tumoroids” emphasizes their direct derivation from GB tumor tissue, and we avoid the use of the term “organoid” because of its association with “organ”, which typically denotes a complex, functional structure composed of multiple tissues that perform specific physiological functions critical to the survival of an organism. In contrast, “brain tumor organoids” are GBCs cultured within brain organoids. This designation emphasizes their growth in a 3D structure resembling a simplified brain-like organ.

To generate acute patient-derived GB tumoroids, tumor tissue was enzymatically dissociated into a single cell suspension as previously described [20, 21]. Tumor tissue was washed with phosphate buffer saline (PBS; #D8537, Sigma), mechanically dissociated using the brain tumor dissociation kit (#130-095-942, Miltenyi Biotec, Bergisch Gladbach, Germany) and gentleMACS dissociator (#130-093-235, Miltenyi Biotec). Dissociated GBCs were resuspended and cultured in ultra-low attachment culture dishes (#CLS3814, Corning, Corning Inc., New York, USA) with Neurobasal-A medium (#10888022, Thermo Fisher Scientific) supplemented with N2 (10 µg/ml; #17502048, Thermo Fisher Scientific), B27 (10 µg/ml), L-glutamine (4 µg/ml; #25030081, Thermo Fisher Scientific), FGF (25 ng/ml) and EGF (25 ng/ml). Cells were cultured for about with medium changes every three days.

Brain organoids were generated from human induced pluripotent stem cells (hiPSCs) as previously described [7]. See Supplementary Material for more details.

To initiate tumor co-culture, brain organoids were each cocultured with 5000 GBCs. After two days, the GBCs growing in cortical spheroids were transferred to standard cell culture dishes precoated with Pluronic (0.5%). After seven days of co-culture, the growth factors EGF and FGF were removed and on day ten the GBCs growing in cortical spheroids were exposed to TTFields. After 5 days of TTFields treatment, GBCs growing in cortical spheroids were fixed and processed.

### In vitro TTFields simulation using inovitro live and inovitro systems

To study the effects of in vitro modeled TTFields on GBC morphology, we used the Inovitro system (Novocure, Haifa, Israel). This device, with ceramic wells, applies alternating current (AC) electric fields to GBCs. Each plate can accommodate up to 8 ceramic wells, allowing different treatment durations synchronized samples. For live Ca^2+^ imaging, we used the Inovitro Live system with plates that have a translucent bottom, enabling time-lapse imaging and real-time quantitative analysis during continuous AC electric field application. Both devices use two perpendicular pairs of electrodes on the outer ceramic wall to model TTFields, with the cell culture dish connected to an electric field generator. To offset heat generated by the electric field, the plate temperature is maintained at 37 °C by placing the dish in a cooling incubator (set at 25 °C; #CCL-170B-8-P, Esco Lifesciences, Friedberg, Germany). The temperature is continuously monitored by two thermistors attached to the walls of the ceramic dish. The intensity of the applied electric field is proportional to the set ambient temperature and is expected to be in the clinically relevant range at approximately 1.12 V/cm when 200 kHz AC is applied [22].

Control experiments were cultured in the same dishes with media changes at the same frequency as the experimental samples. These control samples were maintained in an incubator set at a constant temperature of 37 °C.

To verify the accuracy of the temperature records from the Inovitro hardware and software, manual temperature measurements were taken in the tumor cell media using the Lollipop Traceable Precision Thermometer (#620-2723, Avantor part of VWR International, Pennsylvania, USA). The recorded temperatures were then compared with the values documented by the Novocure Inovitro software.

The Inovitro Live system allows uni-linear electric field orientation to study directional effects. To understand the directional aspects of the electric field, we applied linear AC and assessed the angle at which the TMs aligned relative to the orientation of the applied electric field.

In this study, TTFields experiments were conducted using alternating electric fields at an intermediate frequency of 200 kHz, unless otherwise stated. Previous data indicated a reduced effect at 50 kHz [10], which is why we also used this alternative frequency to confirm these findings.

### Morphological analysis, immunohistochemistry and lentiviral transduction

For morphological analysis of tumor cell networks, 90,000 S24 and T269 GBCs were plated on Matrigel (#356231, Corning Inc.) coated glass coverslips in 24-well plates and maintained in HGM [7].

GBCs were fixed with paraformaldehyde (4% PFA; #P087.1, ROTI Histofix, Roth, Karlsruhe, Germany) for 10 min, washed with Tween 20 (0.5%; #9005-64-5, Sigma) in PBS, stained with Alexa Fluor Phalloidin 546 (#A22283, Molecular Probes, part of Thermo Fisher Scientific) and Hoechst33342 (#H3570, Invitrogen, part of Thermo Fisher Scientific) for 30 min, then mounted (Vectashield Hardset; #H-1400, Vector Laboratories, Newark, California, USA).

Fixed GBCs in organoids were processed, sunk in sucrose (30%; #84097, Sigma) overnight, embedded in 10%/7.5% gelatin/sucrose (gelatin; #9000-70-8, Sigma) and cryosectioned at 20 μm. Samples were blocked in bovine serum albumin (0.5% BSA; #9048-46-8, Sigma) and Triton X-100 (0.1%; #A4975, AppliChem, Darmstadt, Germany), incubated with primary antibodies overnight, washed, incubated with secondary antibodies, counterstained with Hoechst33342 and mounted (Vectashield Hardset).

Primary antibodies used included Nestin (1:600; #MAB1259, Novus Biologicals, part of Bio-Techne, Minneapolis, Minnesota, USA) and Ki67 (1:500, #9129S, Cell Signaling Technology, Danvers, Massachusetts, USA). Caspase (1:500; #ab13847, Abcam, Cambridge, UK). Donkey anti-mouse IgG Alexa Fluor 488 (1:1000; #A-21202, Thermo Fisher Scientific) and goat anti-rabbit IgG Alexa 555 (1:1000; #A-21429, Thermo Fisher Scientific) secondary antibodies were used. Nuclei were stained with Hoechst33342.

For stable fluorescent labeling, GBCs were transduced with lentiviral vectors using pLKO.1-puro non-target shRNA (#SHC016V, Sigma) for cytoplasmic GFP expression. Cells were incubated with lentiviral particles and polybrene (10 μg/ml; #TR-1003-G, Merck, Darmstadt, Germany) for 24 h as previously described [23].

### Quantification of proliferation (Ki67), cell death and infiltration (nestin) in brain tumor organoids

We quantified proliferating cells based on their Ki67 immunofluorescence, cell death based on caspase 3 immunofluorescence and tumor cell infiltration using nestin fluorescence in mature brain tumor organoids (BTO) with Fiji 2.0.0 (RRIDD:SCR_002285). Images were reconstructed into multi-channel 2D maximum intensity projections. The tumor core was defined by dense nestin fluorescence. The Fiji area selection tool measured the overall size of the BTO, the tumor core, and the diffusely infiltrated area. Ki67^+^ cells and caspase 3^+^ cells were manually counted.

Nestin staining quantification was done using a Fiji macro. Two 200 μm square regions within the diffusely infiltrated area were analyzed per BTO. The green nestin channel was isolated from the blue Hoechst33342 channel, and a standard iterative thresholding algorithm was applied. The green channel area was normalized the blue channel area to account for cell number variations.

### Cell viability (PI) and proliferation (EdU) assays in the 2DTM model

We seeded 90,000 S24 GBCs onto Matrigel-coated glass coverslips. Inovitro application was performed for 3 and 5 days. On day 5, cells were exposed to 5-ethynyl-2-deoxyuridine (10 μM EdU) for 3 h and stained using the EdU click assay (#BCK-EdU488, Sigma) and Hoechst33342. For viability assessment S24 GBCs were stained with Hoechst33342 and propidium iodide (1.5 μM PI; #P4170, Sigma). Confocal microscopy was used for imaging. Images were processed with ImageJ, and quantification was performed manually. Proliferation was determined by the ratio of EdU-positive to Hoechst33342-positive nuclei. Dead cells still adhering to the Matrigel matrix were determined as the ratio of PI-positive nuclei to Hoechst33342-positive nuclei.

### Recording of intercellular Ca^2+^ transients

1.5 million S24 or BG5 GBCs were plated in 2DTM culture on a Matrigel-coated polymer coverslip bottom (#81,156, Ibidi, Fisher Scientific) in HGM [7]. After 24 h, cells were exposed to TTFields for 24 h. To assess acute effects, cells were exposed to TTFields after 2 days of culture, and images were acquired within 2 h of exposure. Untreated control GBCs were maintained for 2 days before imaging. Prior to Ca^2+^ transient analysis, cells were loaded with Rhod-2AM (1 μM; #R1244, Sigma) for 30 min.

Acute patient-derived GB tumoroids were also loaded with Rhod-2AM.

Live Ca^2+^ imaging was performed in temperature-controlled media at 37 °C and 5% CO_2_ using confocal microscopy. Each time series lasted 10 min with images acquired at 1.52 s per frame.

### Image processing and analysis

Images were acquired using a ZEISS LSM 710 confocal microscope and ZEISS ZEN software (Zeiss, Germany). Fiji 2.0.0 (RRID:SCR_002285) and Imaris (Bitplane, Zurich, Switzerland) were employed for image processing, including channel subtraction to remove non-specific background signals. Fiji was also used for data analysis, with manual quantification of TM number following established criteria [2, 3, 24].

### Network analysis

Ca^2+^ transient analysis was performed as previously described [7]. In summary, Ca^2+^ transient analysis utilized Fiji to obtain single-cell mean intensity traces, which were smoothed using MATLAB 2020b (MathWorks Inc., Natick, Massachusetts, USA). Peaks and their amplitudes were identified, and cells with periodic Ca^2+^ activity were defined based on specific criteria. Graph theory and cross-correlation analysis were performed using MATLAB, evaluating synchronization between cells and the speed of Ca^2+^ signal propagation [7, 25, 26]. See Supplementary Material for more details.

### Statistical analysis

Image analyses results were imported into SigmaPlot software (Systat Software, Inc., Erkrath, Germany) for statistical assessment. Normality was evaluated using the Shapiro–Wilk test. For normally distributed data, a two-tailed Student’s *t*-test was employed, while non-normally distributed data were analyzed using the Mann–Whitney test. One-way analysis of variance (ANOVA) followed by Dunnett’s test or the Kruskal–Wallis test was utilized for datasets with more than two groups. Statistically significance was considered at p < 0.05.

## Results

### Stability of inovitro temperature measurements within biologically relevant ranges

To ensure the effects on GBCs were due to electric fields and not due to temperature changes, we validated the Inovitro devices’ technical performance by assessing the temperature profile using two methods. The devices have closed-loop temperature control. Over 12 h (200 kHz AC) with measurements every 15 min as shown in Supplementary Fig. 1a, the Inovitro device’s built-in thermosensor recorded an average of 36.7 °C, while manual measurements showed 37.0 °C. Detailed recordings at 3-s intervals revealed occasional single measurement spikes which are likely measurement errors as shown in Supplementary Fig. 1b. A 5-day investigation, mirroring experimental conditions, revealed temperature fluctuations and a steady decrease in current with increasing resistance before each media change as shown in Supplementary Fig. 1c.

### TTFields influence the morphology of the two-dimensional tumor cell network

**Fig. 1:**
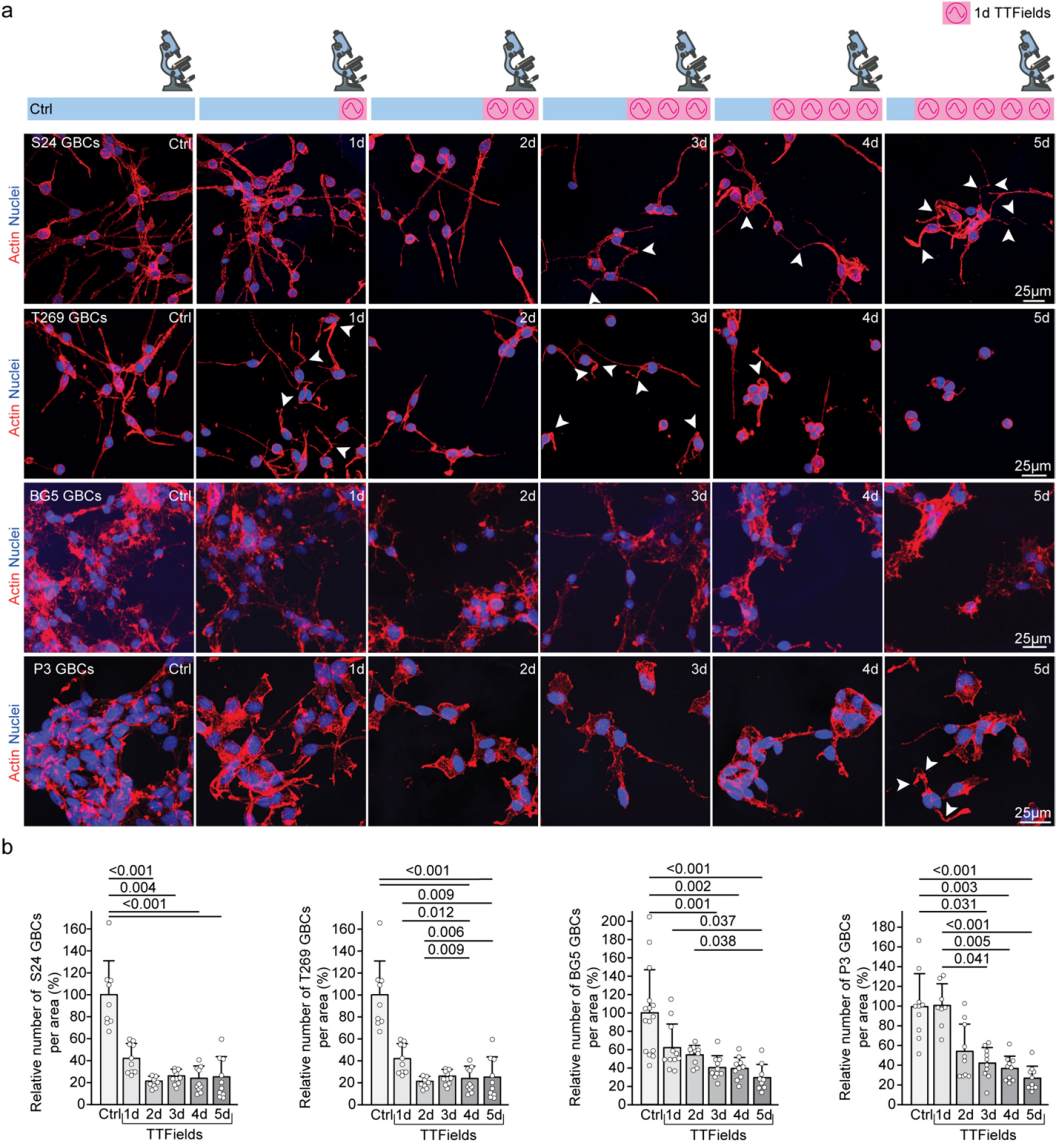
Effect of Inovitro application on glioma network morphology in 2DTM monocultures of GBCs. **a** Cells were either left untreated (control) or subjected to Inovitro-modeled TTFields for 1–5 days. All samples were biologically synchronized to the same age at the end of the experiment. Illustration created using modified bioicons from Servier Medical Art (https://smart.servier.com), licensed under CC BY 4.0. Confocal fluorescence microscopy capturing the effects of Inovitro-modeled TTFields application on S24 (top row), T269, BG5 and P3 (bottom row) GBCs. Phalloidin (binding to F-actin) is shown in red, while Hoechst33342 (binding to adenine–thymine in DNA) is shown in blue. Arrowheads indicate TMs with a unique morphology characterized by an angular change in direction, termed the “crooked TM” phenotype. **b** Examination of the reduction in tumor cell density after application of Inovitro-modeled TTFields. The relative reduction of S24 (left graph), T269, BG5 and P3 (right graph) GBCs per area is shown over 5 days of TTFields exposure compared to the untreated control. Quantification was performed on 8–15 areas from a total of 3 independent samples per time point. Across at all time points, quantification included 275–1150 S24, 120–581 T269, 363–2059 BG5, and 186–378 P3 GBCs from three independent experiments. Mean ± SD, Kruskal–Wallis one-way ANOVA on ranks, Dunn’s method

To elucidate the influence of Inovitro-modeled TTFields on the TM-connected GB cell network, we applied TTFields to a two-dimensional monoculture tumor network (2DTM), which mimics GB morphological and functional in vivo and in patient samples [3, 7, 18]. The experiments used parallel configurations for consistency as illustrated in the cartoons as part of Fig. 1a.

**Fig. 2:**
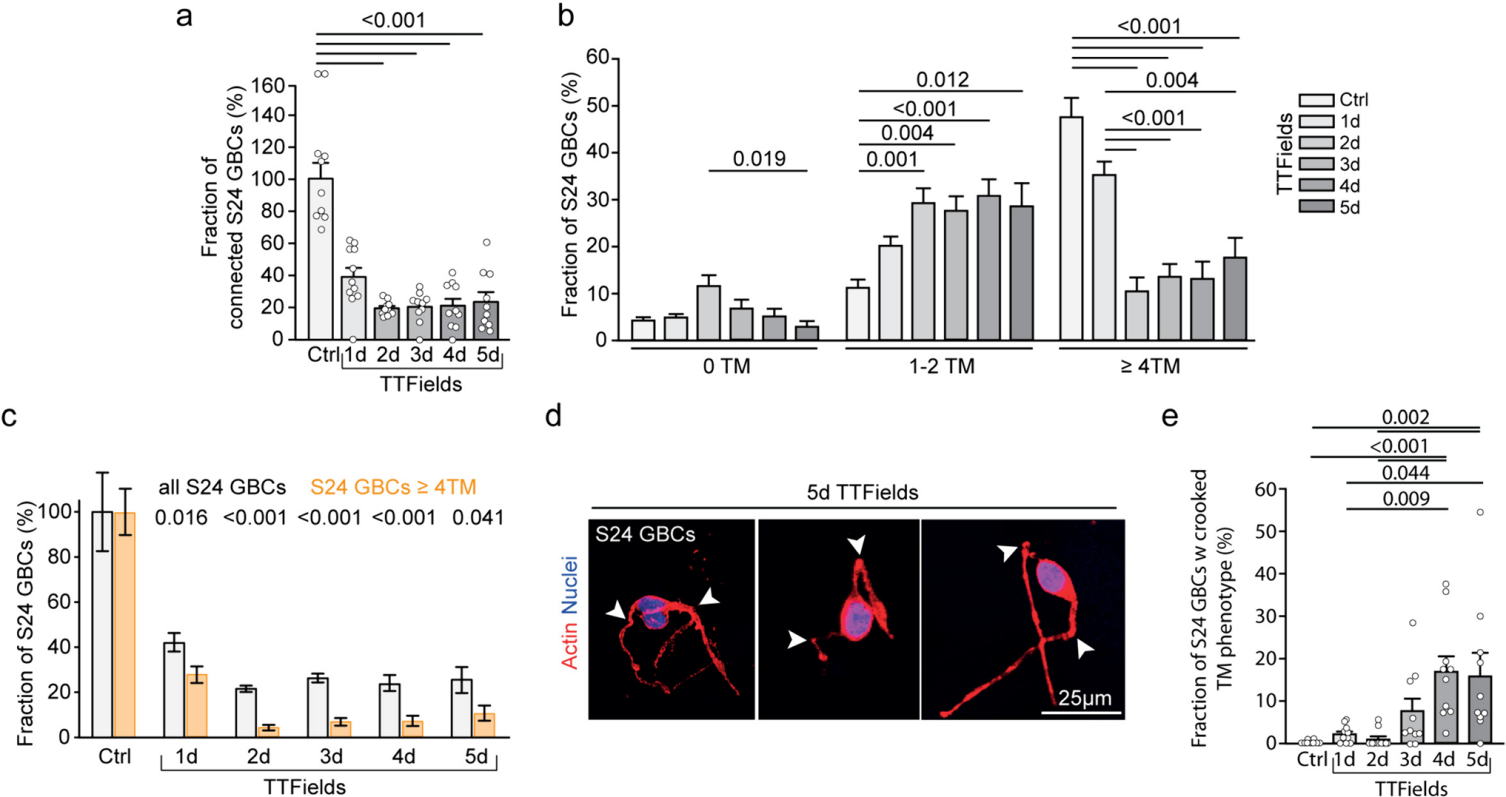
Changes in S24 GBC morphometry when exposed to TTFields **a** Quantification of TM-dependent interconnectivity in TTFields-treated GBCs compared to untreated samples. Quantification included 9–11 areas from a total of 3 independent samples per time point. Mean ± SEM, Kruskal–Wallis one-way ANOVA on ranks, Dunn’s method. **b** Quantitative analysis categorizing S24 GBCs based on the number of TMs at specific time points, highlighting the differential effect of Inovitro-modeled TTField application on different S24 GBC subgroups. Mean ± SD, Kruskal–Wallis one-way ANOVA on ranks, Dunn’s method. **c** Differential effect of Inovitro-modeled TTFields exposure on the morphologically distinct subset of S24 GBCs with ≥ 4TMs (orange columns) compared to all S24 GBCs (gray columns). Mean ± SEM, t-test, Student’s *t*-test. **d**, **e** Identification and quantification of the “crooked TM” phenotype in S24 GBCs treated with TTFields. Mean ± SEM, Kruskal–Wallis one-way ANOVA on ranks, Dunn’s method. **a**–**c**, **e** Across at all time points, quantification included 275–1150 S24 GBCs from three independent experiments. *Ctrl* control, *d* day, *GBC* glioblastoma cell, *TM* tumor microtube

All data refer to 200 kHz TTFields unless otherwise noted. During continuous TTFields, GBC density was reduced to 25% in S24 GBCs, 22% in T269 GBCs, 29% in BG5 GBCs, and 27% in P3 GBCs after 5 days as shown in Fig. 1a, b, leading to a reduction in tumor cell interconnectivity to 24% in S24 GBCs, 11% in T269 GBCs, and to 84% in P3 GBCs, with the network disintegrating over time as shown in Fig. 1a, Fig. 2a and Supplementary Fig. 2a. Morphological network data cannot be quantified in BG5 GBCs growing in the 2DTM model because their tumor microtubes are too intricately intertwined as shown in Fig. 1a.

GBCs show morphologic heterogeneity: some do not project tumor microtubes (TMs), some project one or two TMs, and some are highly interconnected and project multiple TMs [2]. Some also show transient protrusions important for tumor cell migration and scanning of the brain microenvironment known as invadopodia, filipodia, or lamellipodia [27, 28]. The GBC subpopulation with extensive interconnections showed the most response to TTFields, reducing from 53 to 18% in S24 GBCs, from 82 to 9% in T269 GBCs and from 87 to 58% in P3 after 5 days as shown in Fig. 1a, Fig. 2b and Supplementary Fig. 2c. Although GBC numbers decreased, the decline in cells with ≥ 4 TMs was more pronounced as shown in Fig. 2c and Supplementary Fig. 2b.

In vivo*,* TMs are 0.5–2 µm wide, over 500 µm long, and can last over 200 days [2, 17]. In the 2DTM model, TTFields increased the number of curved TMs, termed the “crooked TM” phenotype as indicated by arrows in Fig. 1a, Fig. 2d, e and Supplementary Fig. 2d, e. This phenotype, affecting at least one TM of a GBC, was observed in 0.2% of S24 GBCs and 0.3% of T269 GBCs untreated, rising to 16% in S24 and 6% of T269 GBCs after 5 days of TTFields as shown in Fig. 2e and Supplementary Fig. 2e. The increase in the “crooked TM” phenotype did not reach statistical significance in P3 GBCs.

Experiments using AC in one rather than two perpendicular directions showed no effect of unidirectional electric field orientation on TM as shown in Supplementary Fig. 3a.

TTFields exposure increased the proportion of GBCs undergoing apoptosis from 0.8% in controls to 3.3% after 5 days, assessed by propidium iodide staining as shown in Supplementary Fig. 3b. Cell proliferation is generally low in our 2DTM model and showed no change, as assessed by 5-ethynyl-2-deoxyuridine incorporation when cells were exposed to TTFields as shown in Supplementary Fig. 3c.

In summary, Inovitro-modeled TTFields significantly altered the 2DTM network morphology and interconnectivity, reducing cell density and particularly affecting highly interconnected subpopulations with ≥ 4 TMs, and led to the emergence of a distinct “crooked TM” phenotype.

### TTFields alter network communication patterns in glioma

**Fig. 3:**
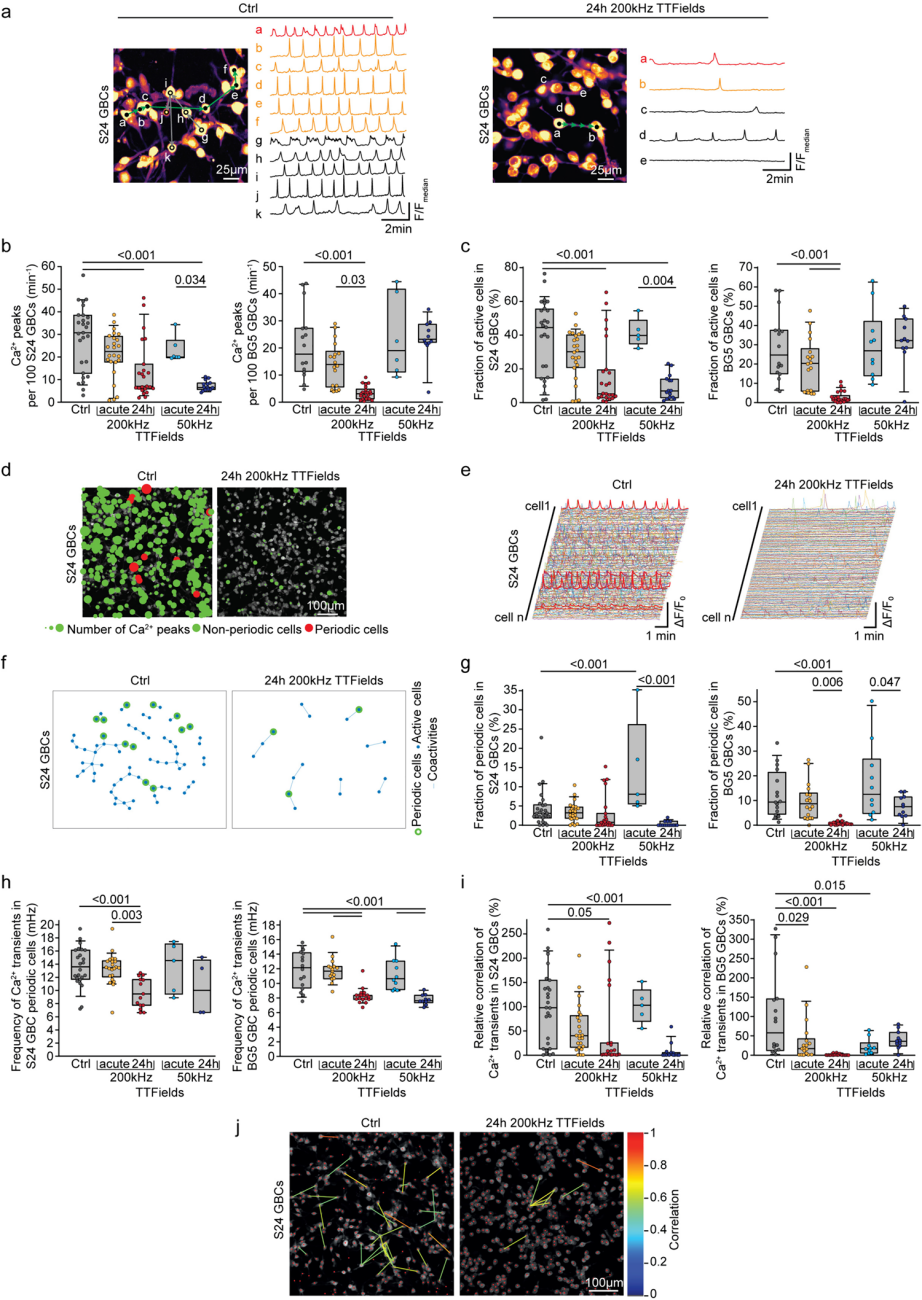
Influence of Inovitro Live-modeled TTFields application on coordinated Ca^2+^ communication patterns in GB tumor cell networks in vitro. **a** Representation of network connectivity between coactive cell pairs extracted from Ca^2+^ recordings of untreated and TTFields-treated S24 GBCs. The direction of Ca^2+^ transients is indicated by arrows; sequential and corresponding traces elicited by a periodic cell ‘a’ are indicated by green arrows. Synchronized Ca^2+^ transients from single cells corresponding to the adjacent network plot are observed in traces a-k in the untreated control and **a**–**e** after 24 h of Inovitro Live-modeled TTFields treatment, respectively. **b** Normalized global Ca^2+^ frequency comparisons for S24 GBCs (left plot) and BG5 GBCs (right plot). **c** Quantification of the fraction of active S24 GBCs (left plot) and BG5 GBCs (right plot). **d** Visualization of representative S24 GBC recordings over a 10 min interval. Red circles represent cells with periodic activity, while green circles represent cells with no or non-periodic Ca^2+^ activity. The size of the circles encodes the frequency of Ca^2+^ peaks in each cell. **e** Ca^2+^ traces from representative S24 GBC recordings without and after 24 h of Inovitro Live treatment, with traces of periodic cells indicated by thick red lines. **f** Example of the functional glioma network derived from S24 GBC Ca^2+^ recordings, including an untreated control and after 24 h of Inovitro Live-modeled TTFields exposure. **g** Proportion of periodic S24 GBCs (left plot) and BG5 GBCs (right plot). **h** Normalized Ca^2+^ frequency in periodic cells, comparisons for S24 GBCs (left plot) and BG5 GBCs (right plot). **i** Relative correlation of Ca^2+^ transients in S24 GBCs (left plot) and BG5 GBCs (right plot). **j** Network plot showing cross-correlation coefficients exceeding the established cut-off, derived from Ca^2+^ recordings from untreated and TTFields-exposed S24 GBCs. **b**, **c**, **g**–**i** Data include untreated controls, immediate effects after acute exposure to Inovitro Live-modeled TTFields, and Ca^2+^ activity recorded after 24 h of exposure to Inovitro Live-modeled TTFields. TTFields were applied at 200 kHz and 50 kHz. Control: n = 16–27 recordings; acute 200 kHz TTFields exposure: n = 18–14 recordings; 24 h of 200 kHz TTFields exposure: n = 22–23 recordings; acute 50 kHz TTFields exposure: n = 5–10 recordings; 24 h of 50 kHz TTFields exposure: n = 11–15 recordings; n = 3 biologically independent experiments each; error bars are SD, one-way ANOVA, Holm-Sidack method. *Ctrl* control, *d* day, *h* hour, *GBC* glioblastoma cell, *ns* not significant (p ≥ 0.05)

The Inovitro Live device allows live imaging and tracking of cellular responses during TTFields application. Multicellular GB networks in vivo show Ca^2+^ transients moving between tumor cells through TMs, creating co-activity patterns [2, 7]. Inovitro Live-modeled TTFields disrupt these patterns as shown in Fig. 3a, Supplementary Fig. 4a and Supplementary movie 1 and 2. After 24 h of exposure to 200 kHz TTFields, global Ca^2+^ activity, measured by Ca^2+^ peaks, was reduced by 51% in S24 GBCs and 83% in BG5 GBCs as shown in Fig. 3b. A similar effect was seen with 50 kHz TTFields in S24 GBCs but not in BG5 GBCs as shown in Fig. 3b, likely due to differences in resistance and Vout between frequencies as shown in Supplementary Fig. 5.

After 24 h of 200 kHz TTFields, the proportion of active cells dropped from 39 to 16% in S24 GBCs and from 28 to 3% in BG5 GBCs as shown in Fig. 3c–f and Supplementary Fig. 4b-d. The 50 kHz TTFields also reduced active cells in S24 but not in BG5 GBCs. There was a decrease in pacemaker-like periodic cells in both S24 and BG5 GBCs as shown in Fig. 3d–g and Supplementary Fig. 4b–d. The activity frequency of periodic cells decreased from 14 to 10 mHz in S24 GBCs and from 12 to 8 mHz in BG5 GBCs after 24 h of TTFields exposure as shown in Fig. 3h. Intercellular co-activity was also reduced as shown in Fig. 3f, i, j and Supplementary Fig. 4d, e.

**Fig. 4:**
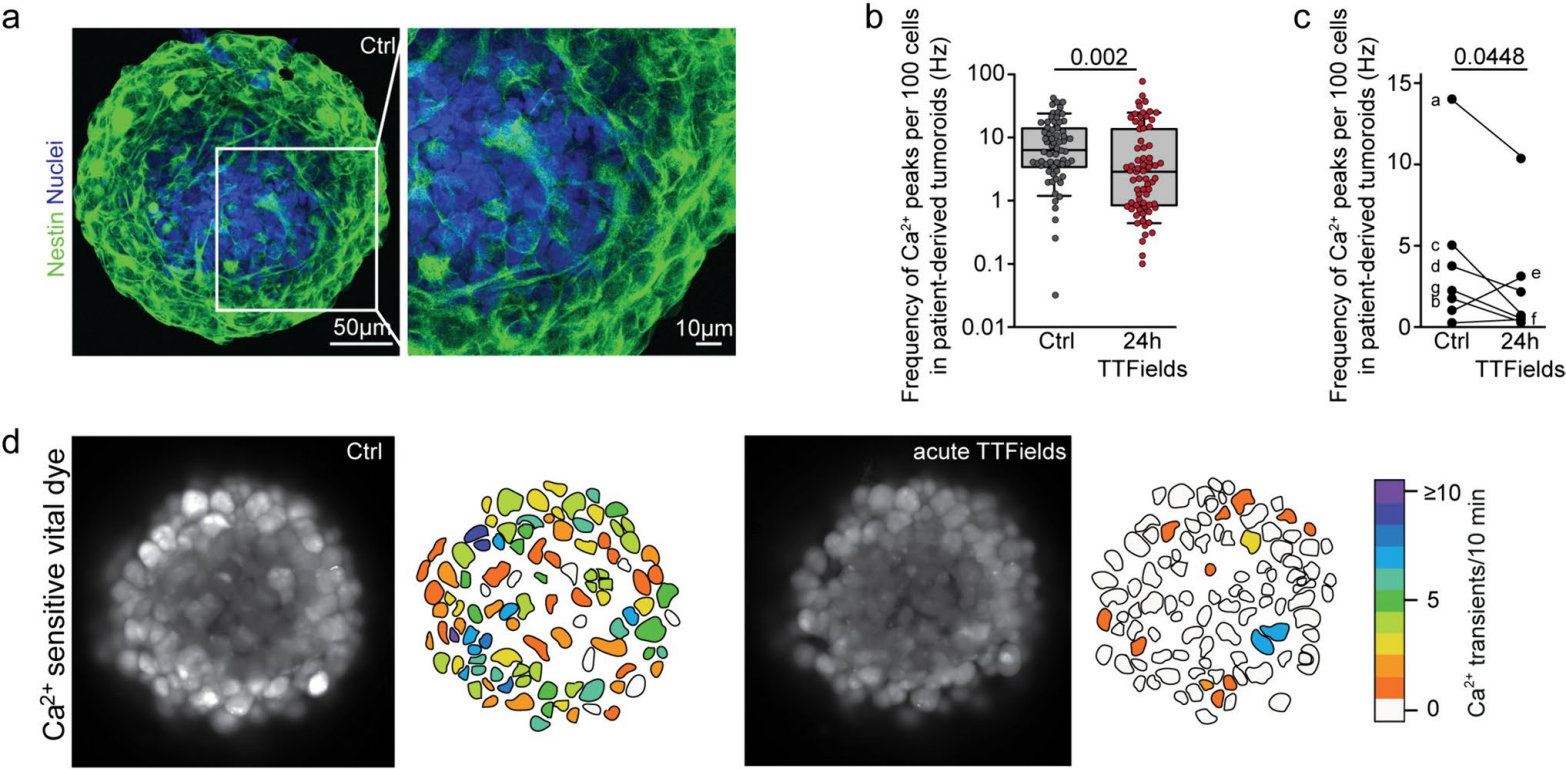
Effect of TTFields on global Ca^2+^ activity in patient-derived tumoroids from freshly resected GB tissue. **a** Patient-derived tumoroids stained with nestin (green) and Hoechst33342 (blue). Tumor cells are interconnected to form a morphologic tumor cell network. **b** Comparison of global Ca^2+^ frequency in untreated control tumoroids and tumoroids exposed to Inovitro Live-modeled TTFields for 24 h. Error bars are SD, t-test, Mann–Whitney rank sum test. **c** Mean global Ca^2+^ activity in untreated and TTFields-treated tumoroids, grouped by parental tumor. Paired *t*-test. **d** Representative Ca^2+^ activity of individual cells within a patient-derived tumoroid before treatment (left panel) and after acute treatment with Inovitro Live-modeled TTFields (right panel). The frequency of Ca^2+^ transients over a 10 min period is color-coded. **b**, **c** Control: recordings from n = 64 tumoroids; 24 h of TTFields exposure: recordings from n = 71 tumoroids; n = 4–12 tumoroids per parental tumor derived from 7 patients. Histologic and molecular characteristics of the parental tumors are listed in Table 1. *Ctrl* control, *h* hour

To avoid model bias and test the effectiveness of TTFields in a 3D model, we used a patient-derived tumoroid platform [21] as shown in Fig. 4a. The molecular specificities of the parental GB tissues are listed in Table 1. After 24 h 200 kHz TTFields, total Ca^2+^ activity reduced in pooled cells from all tumoroids as shown in Fig. 4b, d and Supplementary movie 3, with reductions in 5 out of 7 patients as shown in Fig. 4c.

**Table 1:** Histological and molecular tumor characteristics

Patient ID	Diagnosis	MGMT	Ki67 (%)	Methylation class/CNV
a	Glioblastoma, IDH wt, WHO grade 4	Methylated (13%)	20	Not determined
b	Glioblastoma, IDH wt, WHO grade 4	Methylated (13%)	20	Not determined
c	Glioblastoma, IDH wt, WHO grade 4 (giant cell morphology)	Methylated (54%)	15–20	Not determined
d	Glioblastoma, IDH wt, WHO grade 4	Non-methylated (3%)	10	Mesenchymal / MDM4- Amplification; CDK2A/B-Deletion; loss of Chr: 6q, 9p, 10, 14q, 22q; gain of Chr.:7,
e	Glioblastoma, IDH wt, WHO grade 4	Methylated (16%)	25	Not determined
f	Glioblastoma, IDH wt, WHO grade 4	Non-methylated (1%)	10–20	Mesenchymal / CDK2A/B-Deletion; loss of Chr: 10; gain of Chr.: 7
g	Glioblastoma, IDH wt, WHO grade 4	Methylated	30	RTK II / CDK2A/B-Deletion; loss of Chr. 9, 10; gain of Chr: 9, 20, 21, 7 (incl. EGFR amplification)

Summary of histologic and molecular tumor characteristics for the tissue samples used in Fig. 3

*CDK* cyclin-dependent kinase, *Chr* chromosome, *CNV* copy number variation, *EGFR* epidermal growth factor receptor, *IDH* isocitrate dehydrogenase, *MGMT* O6-methylguanine DNA methyltransferase, *WHO* World Health Organization, *wt* wild-type

In conclusion, TTFields disrupt glioma network communication by reducing global Ca^2+^ activity and intercellular co-activity.

### TTFields alter tumor cell proliferation and infiltration in brain tumor organoids

**Fig. 5:**
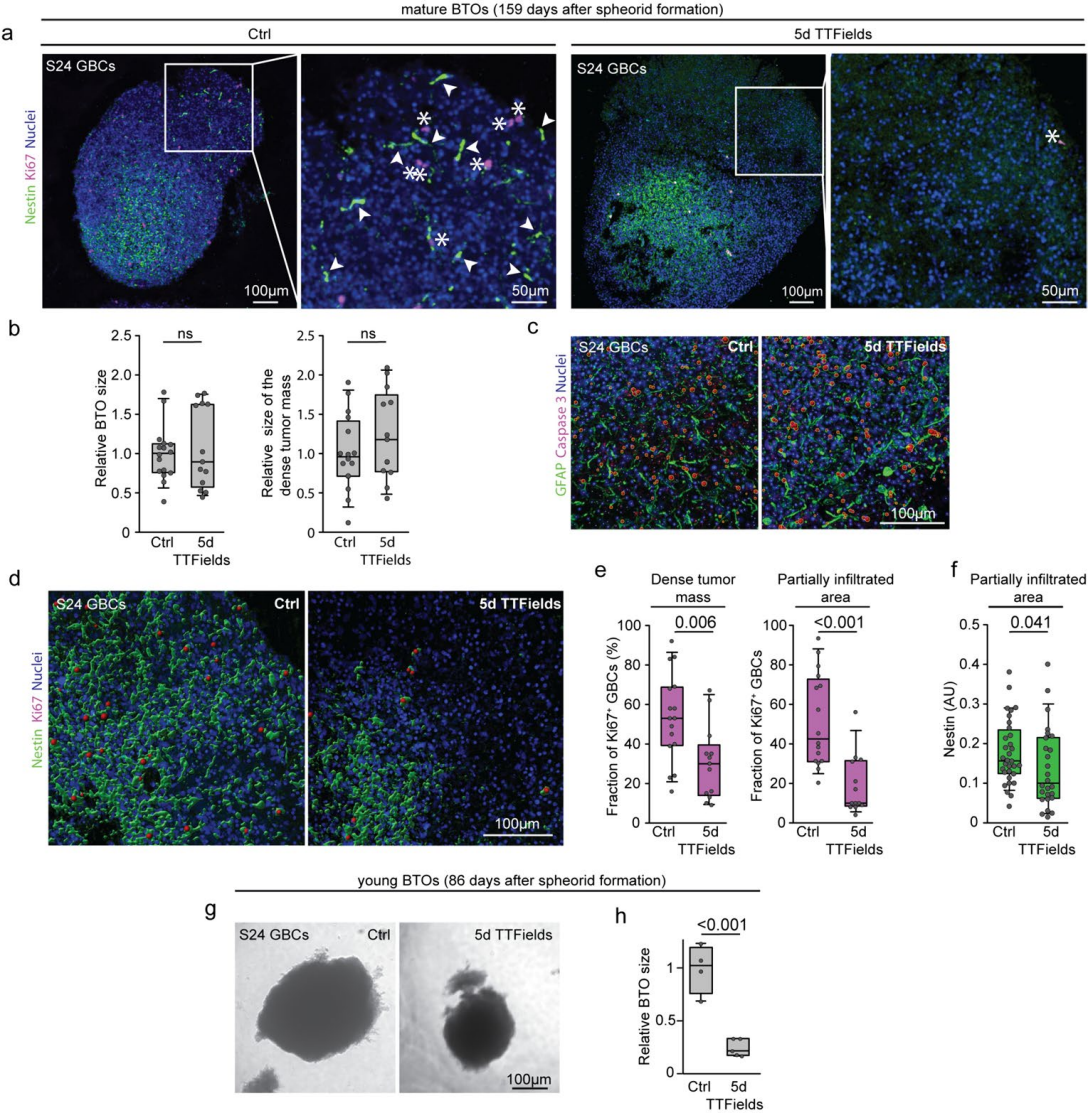
Effect of Inovitro-modeled TTFields on GBCs cultured with mature and young brain organoids. Ki67 and caspase immunofluorescence co-labeling was performed on cryosections of S24 GBC mature brain tumor organoids (BTOs). **a** Untreated S24 GBCs in mature BTOs (left panel) served as a control against mature BTOs exposed to Inovitro-modeled TTFields for 5 days (right panel). The BTOs were 159 days old, with day 1 marking the day of sphere formation. Insets show magnified views of representative areas within the infiltration zone. Nuclei were stained with Hoechst33342 (blue). Arrowheads indicate nestin-stained TMs (green). Asterisks indicate Ki67^+^ nuclei within the diffusely infiltrated area (magenta). **b** BTO size metrics remained statistically indistinguishable between the untreated group (n = 16) and the TTFields-treated cohort (n = 13) for all parameters evaluated. Specifically, no significant differences were observed in overall size (left panel) and in the dimensions of the densely GBC-populated tumor core (right panel) in the mature BTOs, t-test, Welch’s *t*-test. **c** No significant difference was observed in the prevalence of caspase 3 positive nuclei between control and TTField-treated mature BTOs as determined by immunostaining. **d** 3D reconstruction of nestin and Ki67 immunostaining within the area of diffuse tumor cell infiltration in the brain tumor organoid. **e** The number of Ki67^+^ nuclei was significantly reduced in the cohort exposed to Inovitro-modeled TTFields (n = 13) compared to the untreated control (n = 16), both in the tumor core and in the diffusely infiltrated area, as determined by t-test, Student’s *t*-test. **f** The nestin area, normalized to the DAPI area, showed increased nestin levels as an indicator of tumor cell infiltration in the untreated control (n = 16) compared to the cohort exposed to Inovitro-modeled TTFields (n = 13). Quantification was performed in two different regions within the infiltration zone of each BTO. *t*-test, Mann–Whitney rank sum test. **g**, **h** Brain tumor organoids (BTOs) at 86 days of age, day one being the day of sphere formation, showed a significant decrease in overall size when treated with TTFields compared to control samples. TTFields: n = 5, control: n = 4, unpaired t-test. *BTO* brain tumor organoid, *d* day, *GBC* glioblastoma cell

Brain tumor organoids (BTOs) incorporate patient-derived tumor cells into a healthy brain organoid derived from pluripotent stem cells to study tumor-brain interactions, such as infiltration. While the overall size of the mature BTOs and the densely populated tumor core were similar between the TTFields-exposed and the untreated groups as shown in Fig. 5a, b, no difference was observed in the rate of cells undergoing cell death as measured by caspase 3 immunofluorescence shown in Fig. 5c, but a significant decrease in tumor cell proliferation was observed. Specifically, the proportion of Ki67^+^ GBCs decreased from 53 to 30% in the densely populated tumor area and from 51 to 19% in the diffusely infiltrated zone upon exposure to TTFields as shown in Fig. 5a, d, e. Additionally, TTFields treatment reduced total infiltration, as evidenced by a decrease in the nestin-positive area normalized by nuclear Hoechst signal as shown in Fig. 5a, f. In contrast to GBCs growing in mature BTOs, younger BTOs with brain organoids aged 86 days instead of 159 days showed a significant overall decrease in size, beyond a reduction in tumor size as shown in Fig. 5g, h.

In conclusion, TTFields significantly reduce tumor cell proliferation and infiltration in mature BTOs.

## Discussion

Here, we report the first experimental investigation of the effects of TTFields, generated by the Novocure Inovitro devices on GB tumor cell networks. The effects of TTFields on TMs, TM networks, and TM network communication and activation will provide the basis for further investigation in an in vivo setting.

Utilizing various model systems, such as the two-dimensional in vitro monolayer (2DTM), patient-derived three-dimensional tumoroids [21], and GBCs growing in brain organoids, our investigations have revealed the principles and biological significance of glioma network communication [2, 3, 7]. In each system, GBCs cooperate to form a robust, interconnected tumor cell network [2, 7, 27].

In our 2DTM model, we observed a reduction in tumor cell density when exposed to TTFields, coinciding with a switch to unconnected “1–2 TM GBCs”, which share both phenotypic and molecular similarities with neural precursor cells (NPCs) and oligodendrocyte precursor cells (OPCs) [27, 29]. This transition may contribute to increased distant recurrence in GB patients treated with TTFields, while maintaining local control in a subset of patients [30]. Glioma cell infiltration on the other hand was significantly reduced in brain tumor organoids, consistent with previous reports [31].

The pivotal role of Ca^2+^ transients in glioma network communication is evident in the pacemaker-like periodic cells with oscillatory Ca^2+^ patterns [7]. Our in vitro models validate the stability and reproducibility of Ca^2+^ transients and multicellular communication [7, 21].

TTFields reduce global Ca^2+^ activity, correlating with reduced proliferation rates and decreased GB network viability [7]. We observed transient temperature changes, significant heating effects are not detected, which is consistent with previous reports [32].

The effects of TTFields initially associated with mitotic disruption, extend to DNA damage response, replication stress, endoplasmic reticulum stress, membrane permeability, autophagy, and the immune response [33]. TTFields may affect tumor cells at the biophysical level, altering the frequency-dependent MAPK and NF-ĸB pathways [34]. This hypothesis is supported by recent data showing TTFields-induced downregulation of NF-ĸB signaling in vitro [35].

Theoretical models suggest that cells with a narrow mitotic furrow during telophase/cytokinesis are more sensitive to TTFields. The three-dimensional tumor network induces a non-uniform electric field, contributing to the “crooked TM” phenotype.

TTFields may induce changes in intercellular Ca^2+^ transients through oscillatory ionic currents, effecting motor protein movement, cytoskeletal architecture, gap junction dynamics, and overall cytoplasmic charge [36]. These perturbations may culminate in cell death. However, questions remain regarding the specificity of TTFields on tumor cells and sparing of healthy brain cells.

In conclusion, our in vitro studies have provided insights into the effects of TTFields on GB networks. To determine the translational impact, in vivo experiments with tumors in their physiological brain environment and exposed to TTFields power densities similar to the human exposure are imperative. These in vivo studies will be critical in demonstrating the mechanisms of TTFields in GB treatment and potentially other cancer types.

## Supplementary Information

Below is the link to the electronic supplementary material.

Supplementary file1 (TIF 7031 KB)

Supplementary file2 (TIF 15428 KB)

Supplementary file3 (TIF 10183 KB)

Supplementary file4 (TIF 15451 KB)

Supplementary file5 (TIF 3546 KB)

Supplementary file6 (DOCX 51745 KB)

Supplementary file7 (MP4 1312 KB)

Supplementary file8 (MP4 2519 KB)

Supplementary file9 (MP4 599 KB)

## Data Availability

No datasets were generated or analysed during the current study.
