# Supplementary figures and images for: Disrupting glioblastoma networks with tumor treating fields (TTFields) in in vitro models
Source: J Neurooncol. 2024 Aug 1;170(1):139–51. doi: 10.1007/s11060-024-04786-0 (PMC11457690; doi:10.1007/s11060-024-04786-0)

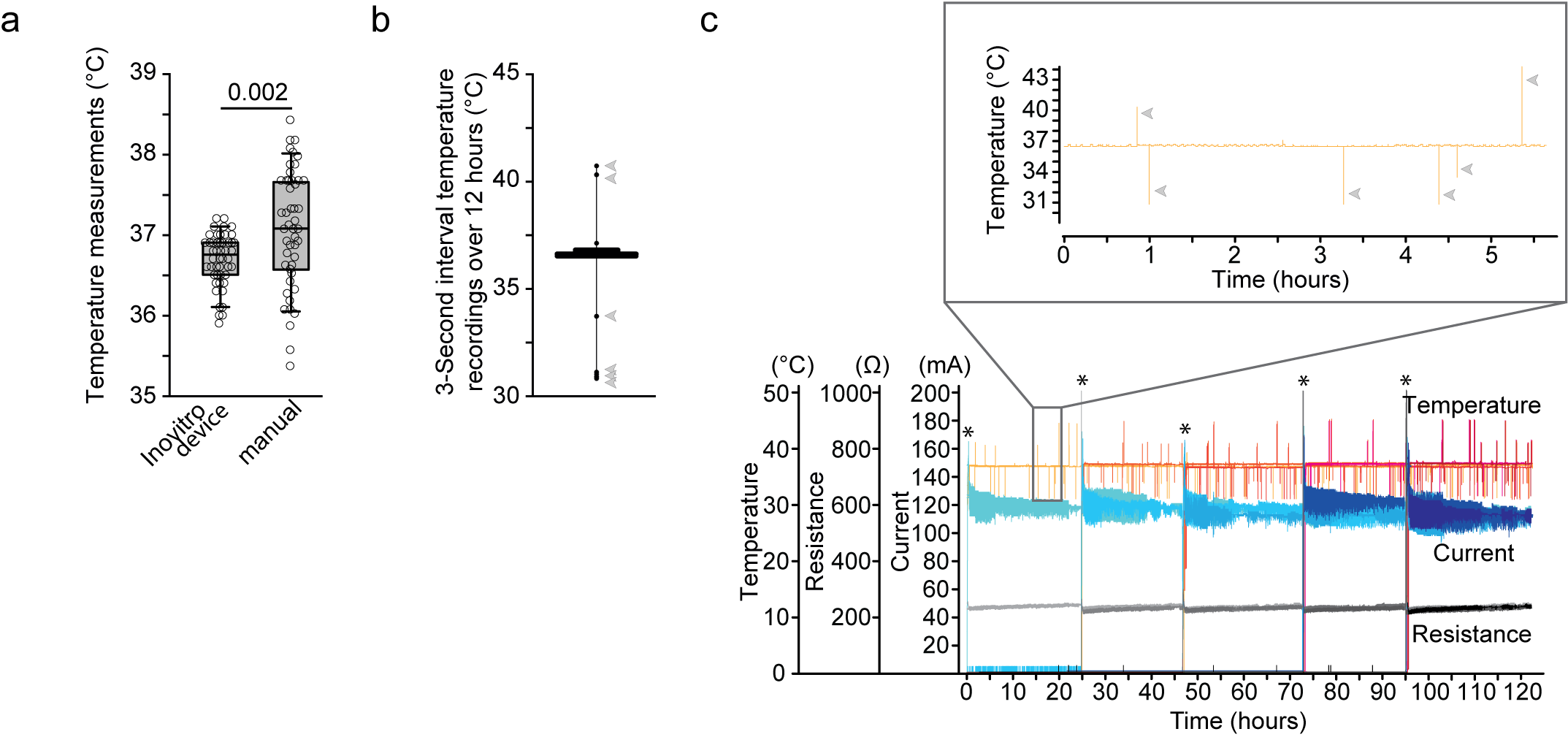

Supplement: Supplementary file 1 — Supplementary file1 (TIF 7031 KB) [file 11060_2024_4786_MOESM1_ESM.tif]

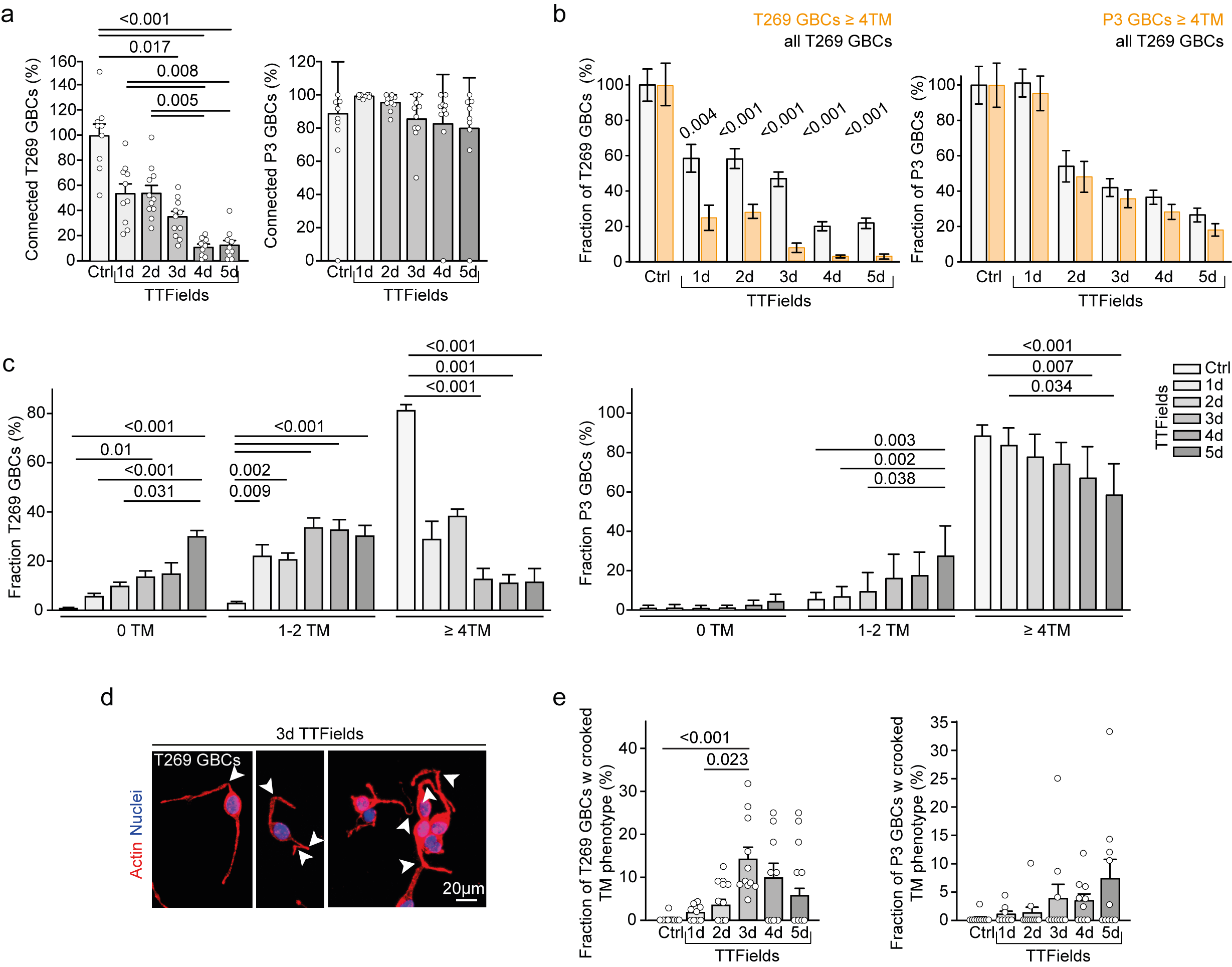

Supplement: Supplementary file 2 — Supplementary file2 (TIF 15428 KB) [file 11060_2024_4786_MOESM2_ESM.tif]

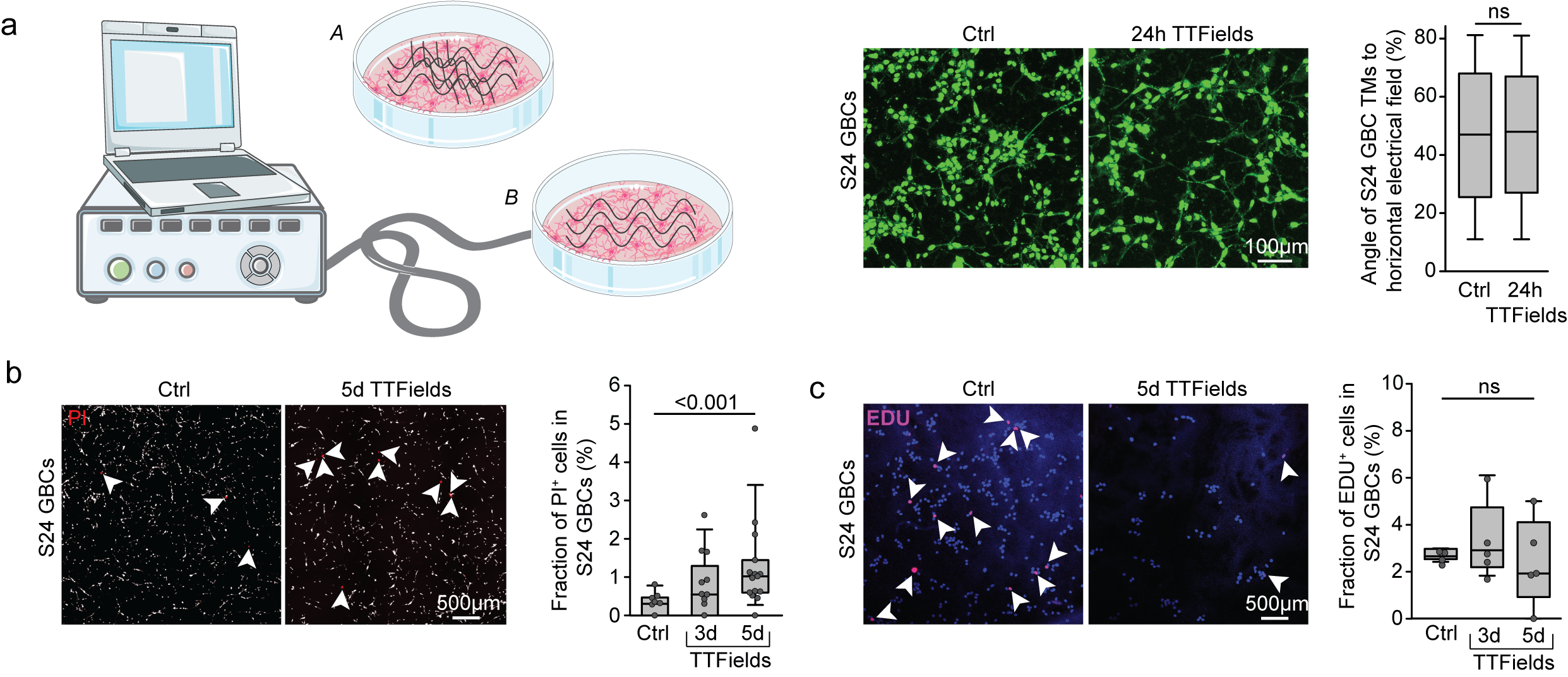

Supplement: Supplementary file 3 — Supplementary file3 (TIF 10183 KB) [file 11060_2024_4786_MOESM3_ESM.tif]

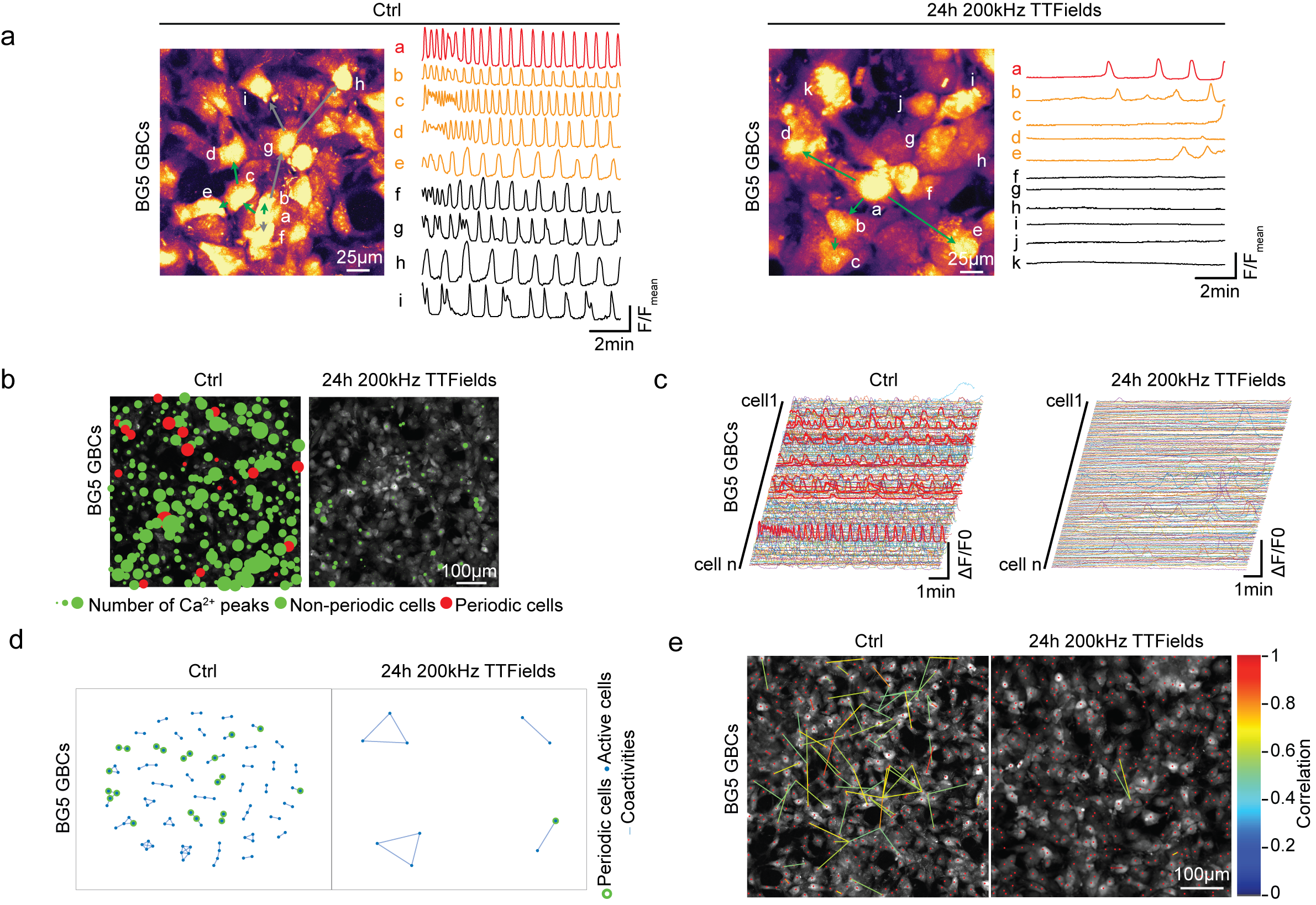

Supplement: Supplementary file 4 — Supplementary file4 (TIF 15451 KB) [file 11060_2024_4786_MOESM4_ESM.tif]

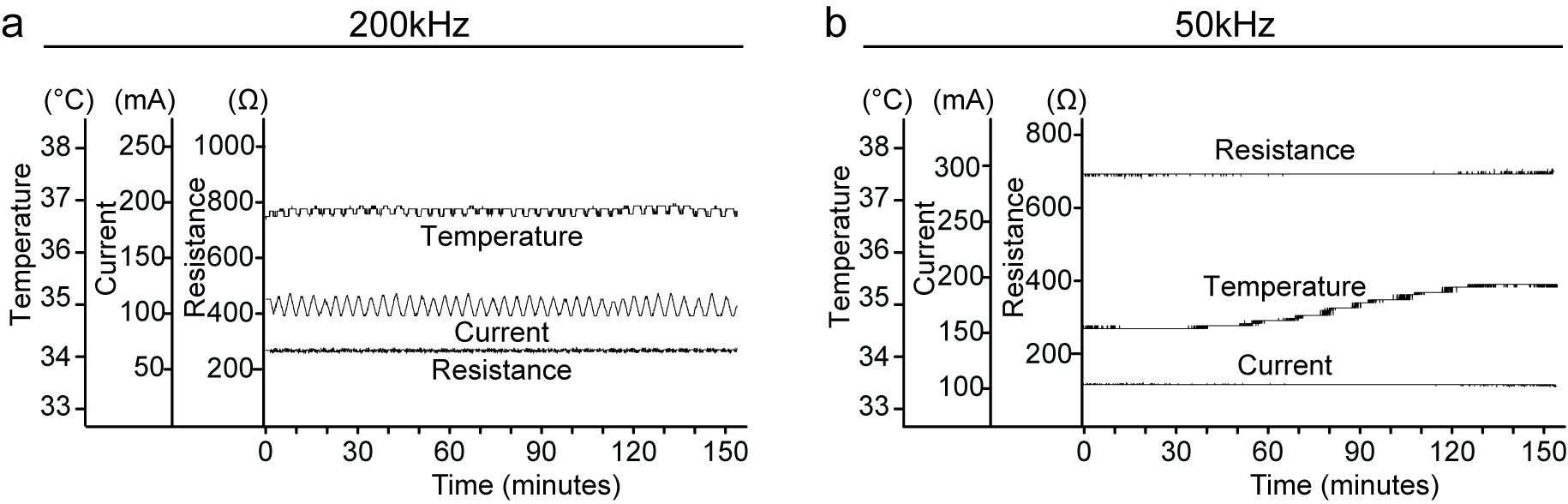

Supplement: Supplementary file 5 — Supplementary file5 (TIF 3546 KB) [file 11060_2024_4786_MOESM5_ESM.tif]
