# Supplementary material for: Disrupting glioblastoma networks with tumor treating fields (TTFields) in in vitro models
Source: J Neurooncol. 2024 Aug 1;170(1):139–51. doi: 10.1007/s11060-024-04786-0 (PMC11457690; doi:10.1007/s11060-024-04786-0)
Supplement: Supplementary file 6 — Supplementary file6 (DOCX 51745 KB) [file 11060_2024_4786_MOESM6_ESM.docx]

Supplementary File

Journal of Neuro-Oncology

**Disrupting Glioblastoma Networks with Tumor Treating Fields (TTFields) in *In Vitro* Models**

Steffen Schlieper-Scherf, Nils Hebach, David Hausmann, Daniel D. Azorín, Dirk C. Hoffmann, Sandra Horschitz, Elena Maier, Phillip Koch, Matthia A. Karreman, Nima Etminan, Miriam Ratliff

Correspondence to: Miriam Ratliff; miriam.ratliff@umm.de; Department of Neurosurgery, University Hospital Mannheim, University of Heidelberg, Germany

**This supplementary file includes**

**Supplementary Figures and Movies**

Supplementary Fig. 1 Stability assessment of the Inovitro (Novocure) hardware parameters

Supplementary Fig. 2 Effect of Inovitro-modeled TTFields on glioma network morphology in 2DTM T269 and P3 GBC cultures

Supplementary Fig. 3 Analysis of cellular responses to Inovitro-modeled TTFields exposure in S24 GBC 2DTM culture

Supplementary Fig. 4 Influence of Inovitro Live-modeled TTFields application on coordinated Ca^2+^ communication patterns in BG5 GBC networks

Supplementary Fig. 5 Characteristics of the applied electric field at 200 kHz compared to 50 kHz AC

Caption for Supplementary Movie 1 Calcium dynamics in S24 GBCs using the in vitro 2DTM assay

Caption for Supplementary Movie 2 Calcium dynamics in BG5 GBCs using the in vitro 2DTM assay

Caption for Supplementary Movie 3 Calcium dynamics in a representative patient-derived tumoroid

**Supplementary Methods**

Generation of brain organoids

Network analysis

**
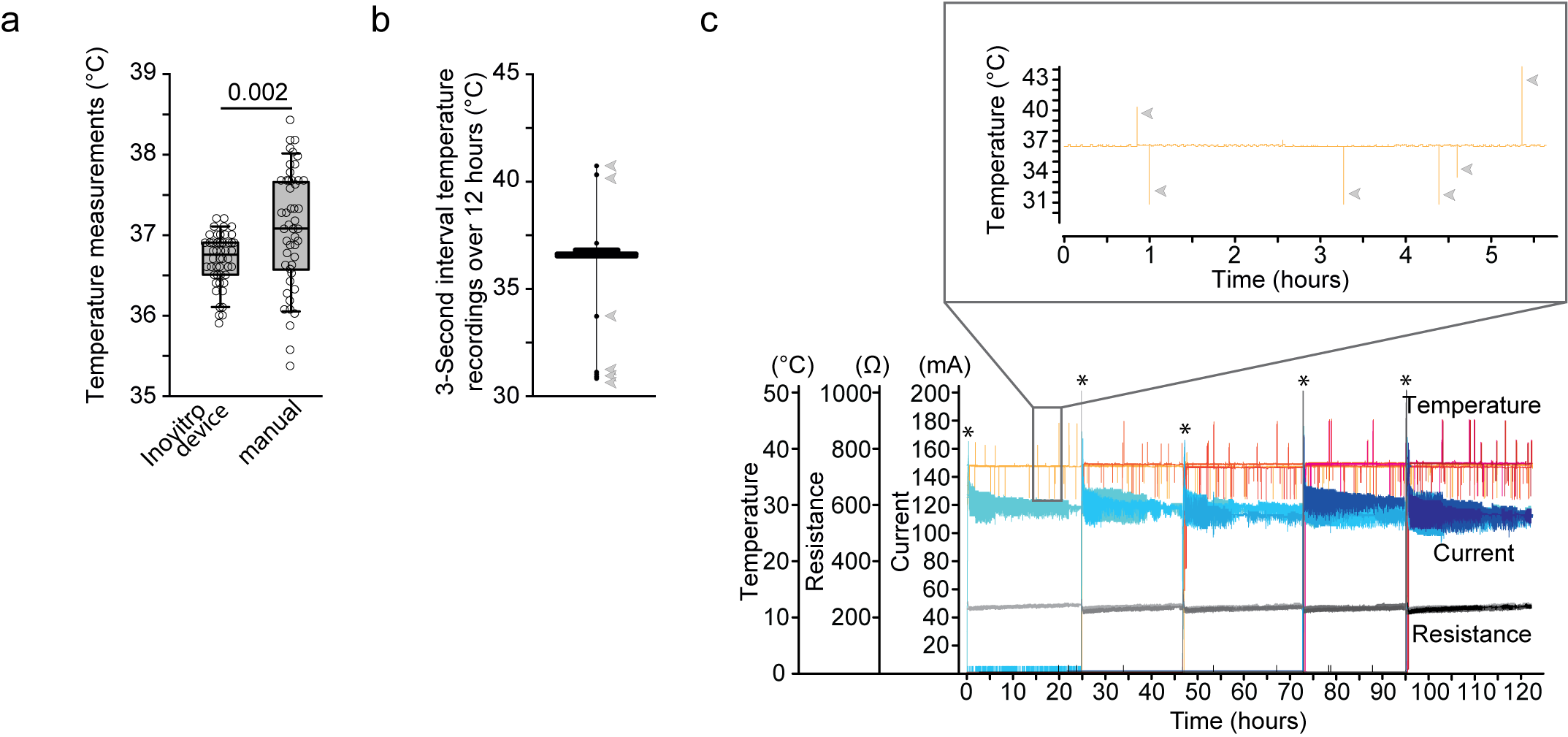
**

**Supplementary Fig. 1** Stability assessment of the Inovitro (Novocure) hardware parameters. **(a)** Comparison of temperature readings obtained from the Inovitro device and temperature readings obtained from a handheld thermometer during a 12-hour time interval. Data points represent measurements taken at regular intervals over the specified time period (48 measurements each, Welch’s t-test) **(b)** The graph shows temperature measurements taken by the Inovitro device at 3-second intervals over a 12 hour period. Each data point represents a temperature measurement taken during the specified time period. **(c)** Representative measurements of temperature (shades of red), current (shades of blue) and resistance (shades of grey) for *in vitro* modeled TTFields using the Inovitro device for 5 days (lightest color), 4 days, 3 days, 2 days and 1 day (darkest color). Asterisks indicate daily media changes. All measurements are superimposed for clarity. The inset image provides a zoomed-in view of the temperature recording. **(b, c)** Arrowheads mark data points that are potentially erroneous.

**
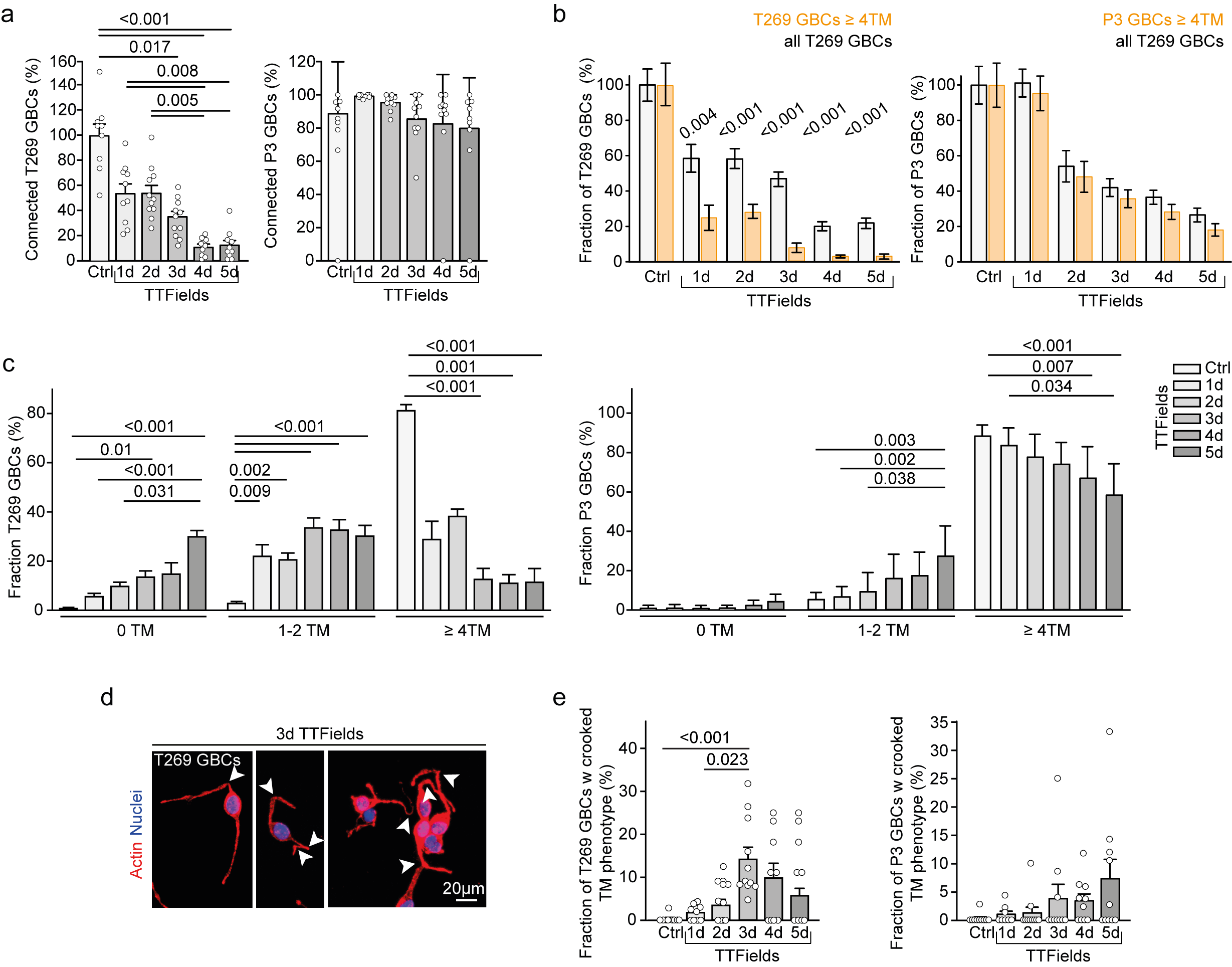
**

**Supplementary Fig. 2** Effect of Inovitro-modeled TTFields on glioma network morphology in 2DTM T269 and P3 GBC cultures. **(a)** Quantification of TM-dependent interconnectivity in T269 (left panel) and P3 (right panel) GBCs exposed to Inovitro-modeled TTFields compared to the corresponding untreated samples. Quantification included 9-11 areas from a total of 3 independent samples per time point. Mean ± SEM, Kruskal-Wallis one-way ANOVA on ranks, Dunn’s method. **(b)** Differential effect of Inovitro-modeled TTFields treatment on the morphologically distinct subset of T269 and P3 GBCs with ≥ 4TMs (orange column) compared to all GBCs (gray column). Mean ± SEM, t-test, Student’s t-test. **(c)** Quantitative analysis categorizing T269 and P3 GBCs based on the number of TMs at specific time points, highlighting the differential effect of TTFields application on different morphologic T269 GBC subgroups. Mean ± SD, Kruskal-Wallis one-way ANOVA on ranks, Dunn’s method. **(d-e)** Identification and quantification of the “crooked TM” phenotype in T269 and P3 GBCs exposed to TTFields. Mean ± SEM, Kruskal-Wallis one-way ANOVA on ranks, Dunn’s method. **(a-c, e)** Across all time points, quantification included 120-581 T269 and 186-378 P3 GBCs from three independent experiments. Abbreviations: Ctrl, control; d, day; GBC, glioblastoma cell; mHz, millihertz; TM, tumor microtube.

**
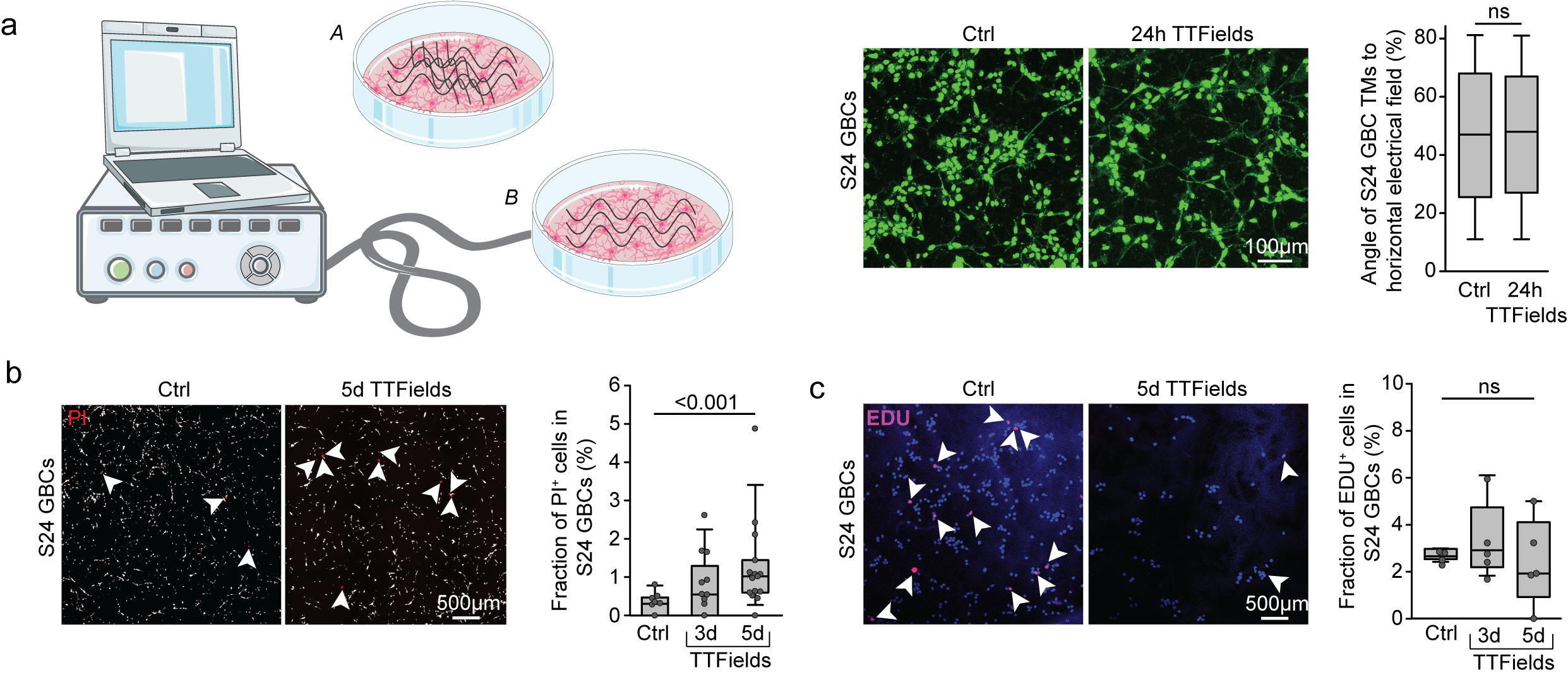
**

**Supplementary Fig. 3** Analysis of cellular responses to Inovitro-modeled TTFields exposure in S24 GBC 2DTM culture. **(a)** Schematic illustration showing that while in all other experiments AC was applied in two perpendicular directions *A*, in this subfigure we analyzed the angles of the TMs relative to the linear electric field with alternating electric field vector direction *B* (200 kHz TTFields). Angle measurements were performed 24 hours after the start of GBC monolayer culture in untreated GBCs and in GBCs exposed to Inovitro Live-modeled TTFields for 24 hours. Angles were measured in n = 1257 TMs of untreated S24 GBCs and in n = 1181 TMs of S24 GBCs exposed to TTFields for 24 hours from a total of 3 independent samples each. t-test, Mann-Whitney rank sum test. **(b)** Number of PI-positive cells divided by Hoechst33342-positive cells in control and S24 GBC monolayer cultures exposed to Inovitro-modeled TTFields. Representative images of untreated PI-stained S24 GBCs and S24 GBCs exposed to Inovitro-modeled TTFields for 5 days. The corresponding box blot shows the relative number of PI-positive untreated control GBCs and GBCs after 3 and 5 days of Inovitro-modeled TTFields application, respectively. Quantification included 10-15 areas from a total of 3 independent samples per time point. Kruskal-Wallis one-way ANOVA on ranks, Dunn’s method. **(c)** Number of EdU-positive cells divided by Hoechst33342-positive cells in control and TTFields-exposed S24 GBCs in 2DTM culture. Representative images of untreated EdU-stained S24 GBCs and S24 GBCs exposed to TTFields for 5 days. The corresponding plot shows the relative number of EdU-positive GBCs in the control and after 3 and 5 days of TTFields exposure, respectively. Quantification included 5 areas from a total of 3 independent samples per time point. Kruskal-Wallis one-way ANOVA on ranks. Abbreviations: Ctrl, control; EdU, 5-ethynyl-2-deoxyuridin; GBC, glioblastoma cell; h, hour; ns, not significant (p ≥ 0.05); PI, propidium iodine; TM, tumor microtube.

**
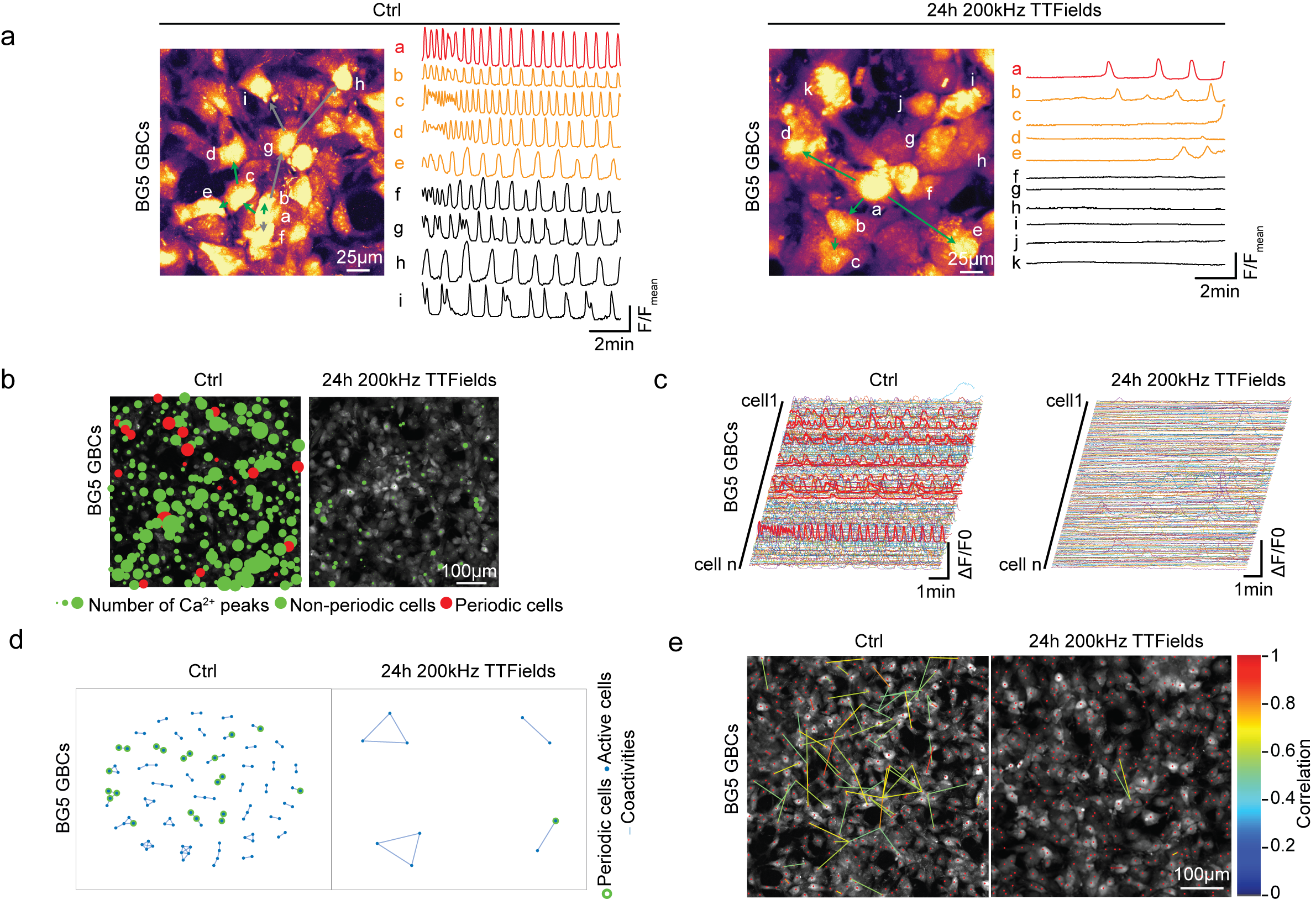
**

**Supplementary Fig. 4** Influence of Inovitro Live-modeled TTFields application on coordinated Ca^2+^ communication patterns in BG5 GBC networks. **(a)** Representation of network connectivity between coactive cell pairs extracted from Ca^2+^ recordings of untreated control BG5 GBCs and TTFields-exposed BG5 GBCs. The direction of Ca^2+^ transients is indicated by arrows; sequential and corresponding traces elicited by a periodic cell ‘a’ are indicated by green arrows. Synchronized Ca^2+^ transients from single cells corresponding to the adjacent network plot are observed in traces a-i in the untreated control and a-k after 24 hours of TTFields exposure, respectively. **(b)** Visualization of representative BG5 GBC recordings over a 10 min interval. Red circles represent cells with periodic activity, while green circles represent cells with no or non-periodic Ca^2+^ activity. The size of the circles encodes the frequency of Ca^2+^ peaks in each cell. **(c)** Ca^2+^ traces from representative BG5 GBC recordings without and after 24 hours of exposure to Inovitro Live-modeled TTFields, with traces of periodic cells indicated by thick red lines. **(d)** Example of the functional glioma network derived from BG5 GBC Ca^2+^ recordings, including untreated control and after 24 hours of TTFields exposure. **(e)** Network plot showing cross-correlation coefficients exceeding the established cut-off, derived from Ca^2+^ recordings of untreated control and BG5 GBCs exposed to Inovitro Live-modeled TTFields. Abbreviations: Ctrl, control; h, hour; GBC, glioblastoma cell; ns, not significant (p ≥ 0.05).


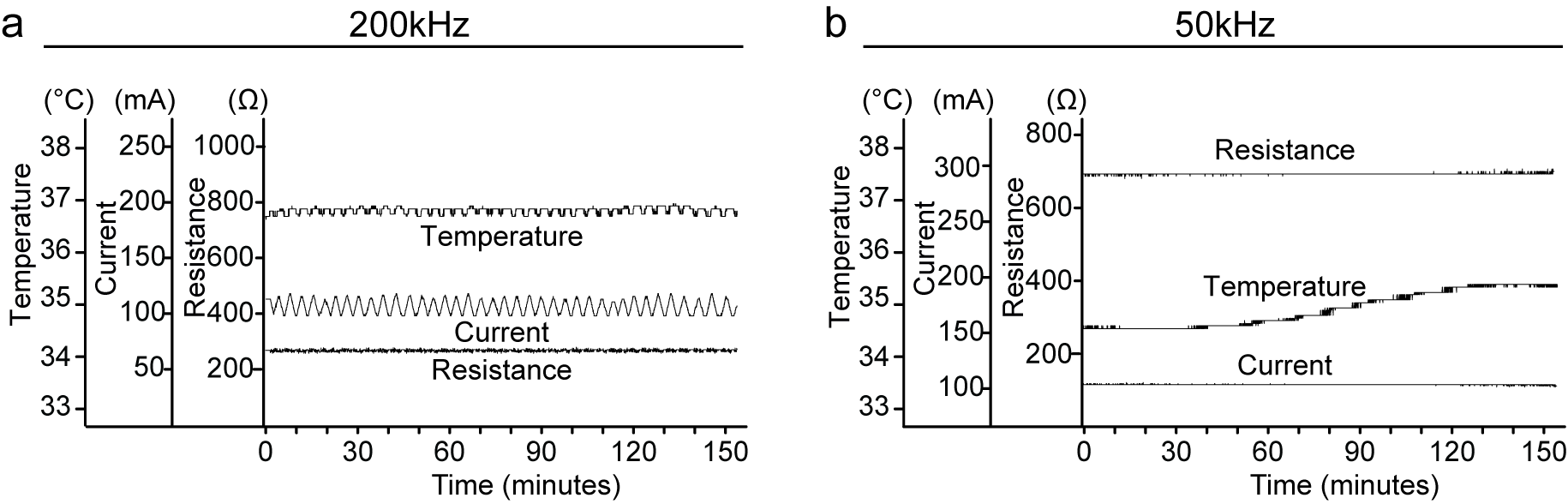


**Supplementary Fig. 5** Characteristics of the applied electric field at 200 kHz compared to 50 kHz AC **(a, b)**. When exposed to 200 kHz TTFields **(a)**, the electrical properties showed significant differences compared to those induced by a 50 kHz electric field **(b)**. The mean resistance at 200 kHz TTFields was 22 Ω, in sharp contrast to 692 Ω at 50 kHz TTFields (p < 0.001). In addition, the mean temperature achieved at 200 kHz TTFields was 36.7°C, whereas it was recorded at 34.8°C with a gradual temperature increase over time in the 50 kHz TTFields (p < 0.001). In addition, Vout was 22 V for the 200 kHz AC model compared to 72 V for the 50 kHz TTFields model. The difference in resistance is expected due to the capacitive nature of the ceramic, as the resistivity of capacitors is inversely related to frequency. (Quantification included 3052-3054 measurements, t-test, Mann-Whitney rank sum test).

**Supplementary Movie 1** Calcium dynamics in S24 GBCs using the *in vitro* 2DTM assay. This video provides an insight into the intricate Ca^2+^ dynamics within S24 GBCs as visualized by the application of the Rhod-2AM Ca^2+^ sensor and time-lapse imaging using confocal microscopy. The sequences presented in this video provide a visual representation of the spontaneous rhythmic Ca^2+^ fluctuations in an untreated control sample (shown on the left), juxtaposed to a biologically identical sample after 24 hours of Inovitro Live-modeled TTFields exposure (shown on the right). The time-lapse images were captured over a period of 10 min and the video has been sped up to run at 20x speed to improve the clarity and efficiency of the data presentation.

**Supplementary Movie 2** Calcium dynamics in BG5 GBCs using the *in vitro* 2DTM assay. This video provides an insight into the intricate Ca^2+^ dynamics within BG5 GBCs as visualized by the application of the Rhod-2AM Ca^2+^ sensor and time-lapse imaging using confocal microscopy. The sequences presented in this video provide a visual representation of the spontaneous rhythmic Ca^2+^ fluctuations in an untreated sample (shown on the left), juxtaposed to a biologically identical sample after 24 hours of Inovitro Live-modeled TTFields exposure (shown on the right). The time-lapse images were captured over a period of 10 min and the video has been sped up to run at 20x speed to improve the clarity and efficiency of the data presentation.

**Supplementary Movie 3** Calcium dynamics in a representative patient-derived tumoroid. This video provides an insight into the TTField effect on the intricate Ca^2+^ dynamics within a patient-derived tumoroid as visualized by the application of the Rhod-2AM Ca^2+^ sensor and time-lapse imaging using confocal microscopy. The sequences presented in this video provide a visual representation of the spontaneous rhythmic Ca^2+^ fluctuations in an untreated control tumoroid sample (shown on the left), juxtaposed to the identical tumoroid sample after acute exposure to Inovitro Live-modeled TTField (shown on the right). The time-lapse images were captured over a period of 10 min and the video has been sped up to run at 20x speed to improve the clarity and efficiency of the data presentation.

**Supplementary Methods**

**Generation of brain organoids**

To generate brain organoids, neural progenitor cells (NPCs) were isolated as single cells using TrypLE Express (#12604013, Thermo Fisher Scientific) and seeded at a density of 100,000 cells per well in V-bottom 96-well plates (#277143, Thermo Fisher Scientific) coated with Pluronic F-127 (0.5%; #9003-11-6, Sigma). The culture medium was supplemented with ROCK inhibitor Y-27632 (50 µM; #146986-50-7, Cell Guidance Systems Ltd). After two days, the resulting spheroids were transferred to 6 cm culture dishes pre-coated with Pluronic F-127 (0.5%). After five days, the small molecules were removed and the medium was supplemented with EGF (20 ng/ml) and FGF (20 ng/ml) for the next 19 days, with regular medium changes every other day. On day 27, the growth factors FGF and EGF were removed from the culture medium and neuronal maturation was promoted by supplementing the medium with ascorbic acid (200 μM; #A4544, Sigma), LM22A (1 μM; #SML0848-25MG, Sigma), LM22B (1 μM; #6037, Tocris Bioscience, part of Bio-Techne, Bristol, UK), PD-0332991 (2 μM; #1116, Selleck Chemicals, Cologne, Germany), DAPT (5 μM; #SM15-50, Cell Guidance Systems Ltd), CHIR99021 (3 μM; #SM13-50, Cell Guidance Systems Ltd), forskolin (10 μM; #SM18-100, Cell Guidance Systems Ltd), and GABA (300 μM; #A5835-10G, Sigma). The medium was changed twice a week. After 4 days, the medium was changed to Neurobasal containing 1% B27, 1% penicillin-streptomycin, 1x GlutaMAX, and 5 mg/ml D-glucose. DAPT, forskolin, and GABA were removed at the next medium change and CHIR99021 was withdrawn one week later.

**Network Analysis**

We used Fiji to obtain single-cell mean intensity traces over time. Using MATLAB 2020b (MathWorks Inc., Natick, Massachusetts, USA), we then applied a Gaussian filter to smooth the single-cell traces and identified peaks and their respective amplitudes using a peak finder function. To generate the scrambled data, we rearranged the peaks using circular shift redistribution within each cell, keeping the total number of peaks per cell the same, but randomizing their temporal occurrence.

We established specific criteria for the identification of cells with periodic Ca^2+^ activity. These cells were defined as having a minimum of four Ca^2+^ peaks, and the standard deviation of all peak-peak intervals had to be less than 15 seconds over a 10-minute observation period *in vitro*. To accurately calculate the standard deviation of all peak-peak intervals, a minimum of four peaks (and therefore at least three peak-peak intervals) was considered necessary. Therefore, for subsequent analysis, active cells were characterized as cells with at least four peaks.

Graph theory and cross-correlation analysis were performed using MATLAB, following previously established procedures [1-3]. Briefly, we assessed the synchronization between cells by adjusting pairs of single-cell traces ($a$ and $b$) forward and backward in time relative to each other. This adjustment took into account the time taken for the Ca^2+^ transient to propagate from one cell to the other. The direction in which a single-cell trace had to be shifted to achieve maximum correlation with another single-cell trace provides an insight into the direction of Ca^2+^ signal propagation, distinguishing one cell as the trigger and the other as the receiver. We calculated the speed of the travelling Ca^2+^ signal by taking into account the time shift and the distance between the two cells. Cells with fewer than four peaks, cell pairs separated by more than 100 μm, and correlations indicating signal propagation at speeds < 4 μm/s or > 25 μm/s were excluded to avoid consideration of random correlations. The range of signal velocities was determined by manually measuring the velocity of several Ca^2+^ signals travelling between two cells.

As a negative control, we used a scrambled data set consisting of randomly assembled cell signals over time. A pair of cells from the empirical data was designated as coactive if the maximum correlation coefficient (${\rho\left( a,b \right)}_{max}$) exceeded certain thresholds: 0.47 for S24 and BG5 GBC monolayer *in vitro* culture*,*

The threshold for the measure of intercellular coactivity was derived from previously established data and then determined by dividing the number of empirically identified coactive cell pairs by the number of all coactive cell pairs found in the corresponding scrambled data [3].

**References**

1. Feldt S, Bonifazi P, Cossart R (2011) Dissecting functional connectivity of neuronal microcircuits: experimental and theoretical insights. Trends Neurosci 34: 225-236 doi:10.1016/j.tins.2011.02.007

2. Malmersjo S, Rebellato P, Smedler E, Planert H, Kanatani S, Liste I, Nanou E, Sunner H, Abdelhady S, Zhang S, Andang M, El Manira A, Silberberg G, Arenas E, Uhlen P (2013) Neural progenitors organize in small-world networks to promote cell proliferation. Proc Natl Acad Sci U S A 110: E1524-1532 doi:10.1073/pnas.1220179110

3. Hausmann D, Hoffmann DC, Venkataramani V, Jung E, Horschitz S, Tetzlaff SK, Jabali A, Hai L, Kessler T, Azorin DD, Weil S, Kourtesakis A, Sievers P, Habel A, Breckwoldt MO, Karreman MA, Ratliff M, Messmer JM, Yang Y, Reyhan E, Wendler S, Lob C, Mayer C, Figarella K, Osswald M, Solecki G, Sahm F, Garaschuk O, Kuner T, Koch P, Schlesner M, Wick W, Winkler F (2023) Autonomous rhythmic activity in glioma networks drives brain tumour growth. Nature 613: 179-186 doi:10.1038/s41586-022-05520-4
